# Prognostic Factors for Survival in Adults With Burkitt Lymphoma: A Systematic Review

**DOI:** 10.1002/cam4.70513

**Published:** 2025-01-29

**Authors:** Aythami de Armas‐Castellano, Diego Infante‐Ventura, Tasmania del Pino‐Sedeño, Yadira González Hernández, Raul Quiros, Beatriz León‐Salas, Vincent Ribrag, María M. Trujillo‐Martín, ERN EuroBlood Working Group

**Affiliations:** ^1^ Canary Islands Health Research Institute Foundation (FIISC) Las Palmas de Gran Canaria Spain; ^2^ Evaluation Unit of the Canary Islands Health Service (SESCS) Santa Cruz de Tenerife Spain; ^3^ Network for Research on Chronicity Primary Care, and Health Promotion (RICAPPS) Palma Spain; ^4^ Internal Medicine Department Hospital Costa del Sol Marbella Málaga Spain; ^5^ Hematology Department Institut Gustave Roussy Villejuif France; ^6^ ERN‐EuroBloodNet Hôpital St Louis/Université Paris 7 Paris France

**Keywords:** Burkitt lymphoma, meta‐analysis, non‐Hodgkin's lymphoma, prognosis, survival, systematic review

## Abstract

**Introduction:**

Burkitt lymphoma (BL) is a rare and aggressive subtype of non‐Hodgkin's lymphoma. Several studies have identified prognostic factors (PFs) for disease progression and mortality among adults with BL. However, there is no consensus on risk stratification based on PFs. This study aims to identify, critically assess, and synthesize the available evidence on PFs for survival in adults with BL.

**Methods:**

A systematic review was conducted. Medline, EMBASE, and CENTRAL were searched from inception to February 22, 2022. Randomized or non‐randomized clinical trials and longitudinal observational studies were eligible for inclusion. Reference screening, data extraction, and risk‐of‐bias assessment were conducted independently and in duplicate. Publication bias was examined by visual inspection of funnel plots. Meta‐analyses were conducted when appropriate using Review Manager 5. The certainty of evidence was assessed using GRADE.

**Results:**

The search identified 1119 references. Of these, 76 papers were selected for full‐text assessment and 36 studies (*N* = 10,882) reported in 39 articles were eligible for inclusion. Older age, higher performance status, and central nervous system involvement were associated with poorer overall survival (OS) and progression‐free survival (PFS). Black patients exhibited significantly lower OS and relative survival. Bone marrow involvement and higher albumin levels were associated with poorer OS. Treatment with rituximab, and with methotrexate were associated with better OS and PFS.

**Conclusion:**

This study provides a comprehensive and methodologically rigorous evidence review on PFs in adults with BL. Several significant associations of PFs and survival estimates were observed, therefore, providing data to inform treatment decisions and to improve patient care.

## Abstract 2

This systematic review identifies and assesses several significant prognostic factors for survival estimates in adults with Burkitt lymphoma, providing useful results to inform treatment decisions and improve patient care.

## 1 Introduction

Burkitt lymphoma (BL) is a rare subtype of non‐Hodgkin lymphoma (NHL) characterized by its highly aggressive nature. Three distinct epidemiological subtypes of BL are recognized: endemic, immunodeficiency‐associated (HIV‐BL), and sporadic BL [1]. There are differences in clinical presentation across BL variants. Endemic BL commonly presents as facial tumors, while HIV‐BL typically includes nodal involvement but also has a high frequency of dissemination to extranodal sites. On the other hand, sporadic BL usually manifests as tumors in the abdomen or bone marrow (BM), accounting for 1%–2% of cases in adults in the United States and Western Europe [2]. BL is more prevalent in males than females and generally presents as a rapidly progressive disease [3]. Thus, the majority of BL patients are diagnosed with an advanced clinical stage [4].

The treatment of BL typically involves intensive chemotherapeutic regimes that may have central nervous system (CNS) involvement as the cytotoxic agents used in these treatments can cross the blood–brain barrier [5]. Younger adults are more likely to receive and tolerate potentially intensive treatments such as hyper‐CVAD and CODOX‐M/IVAC. Hence, both age and treatment protocols are considered influential factors affecting the prognosis in BL [6]. Not in vain, intensive chemotherapy protocols are associated with increased toxicities and the emergence of significant side effects [7].

Several studies have identified factors for disease progression and mortality among patients with BL, such as older age, [8] advanced stage of the disease, race/ethnicity, and poor performance status, among others [4, 9, 10]. These prognostic factors are typically considered in clinical practice to guide treatment decisions. However, to the best of our knowledge, no systematic assessment of these factors [8] is currently available, though it is essential for establishing a common understanding of their impact on disease progression and mortality. Therefore, this study aims to identify, critically assess, and synthesize the available scientific evidence on prognostic factors for survival in patients with BL.

## 2 Materials and Methods

This document presents a systematic review conducted to inform evidence‐based recommendations in the European Reference Network (ERN) EuroBloodNet clinical practice guideline (CPG) on BL. We followed the Cochrane Prognosis Methods Group [11] guidance to perform a systematic review of prognosis studies and the Preferred Reporting Items for Systematic Reviews and Meta‐Analyses (PRISMA) statement for reporting it [12]. Our systematic review protocol is registered in the PROSPERO database (reference no. CRD42022342616).

### 2.1 Information Sources and Search Strategy

The authors searched Medline (Ovid platform), EMBASE (Elsevier interface), and Cochrane (CENTRAL) databases from inception until February 22, 2022. The search strategy included both controlled vocabulary and text‐word terms. Searches were restricted to the English language and to only retrieve studies published in the last 15 years (from 2007). The reference lists of all relevant papers were examined to identify possible additional studies meeting the selection criteria. With the same purpose, articles that referenced the included studies were scouted in Google Scholar. Table S1 provides detailed descriptions of the search strategy.

Additionally, references identified for other questions concerning the EuroBloodNet CPG on BL that included relevant information for this work were also considered for selection. Finally, the list of included studies after the selection process was shared with the EuroBlood clinicians participating in this CPG question so they could add any relevant study not retrieved in the electronic searches.

### 2.2 Study Selection and Study Selection Process

Studies were eligible for inclusion if they fulfilled the following criteria:

- *Type of study*: Randomized controlled trials (RTCs), non‐randomized clinical trials (n‐RCTs), and longitudinal observational studies were included.
- *Population*: Studies that evaluated adults aged 18 and older diagnosed with BL were included. Studies in patients with Burkitt‐like lymphoma, unclassifiable and/or aggressive NHL, and any other NHL were excluded.
- *Index prognostic factor*: The presence of any of the following prognostic factors: age, sex, race/ethnicity, HIV, BM involvement, CNS involvement, risk stratification, treatment, albumin, transplantation, comorbidities, and performance status (a measure of disease impact on daily activities) was considered.
- *Comparator*: The absence of each specific prognostic factor.
- *Outcomes*: to be considered for inclusion, studies had to report on overall survival (OS), disease‐free survival (DFS), progression‐free survival (PFS), event‐free survival (EFS), relative survival (RS) defined as the ratio of the proportion of observed survivors in a cohort of cancer patients to the proportion of expected survivors in a comparable set of cancer‐free individuals, or cancer‐specific survival (CSS) defined as the time from either the date of diagnosis or the start of treatment for BL, to the date of death from the disease. Hazard ratios (HRs) were considered as the measure of effect for these outcomes, although odds ratios (ORs) were also considered for complications of cancer when reported.
- *Timing*: Any time after diagnosis. No studies were excluded based on the duration of their follow‐up.
- *Language*: Only studies published in English were included.
- *Publication type*: Only full original publications were considered. Therefore, conference abstracts were excluded.

The study selection process was conducted independently and in duplicate by two reviewers. First, titles and abstracts of all records identified by searches were screened. The full texts of all articles deemed potentially relevant were then assessed for inclusion to confirm eligibility according to the pre‐determined criteria. The reviewers compared results and discussed doubts and disagreements in both phases, consulting a third reviewer when necessary.

### 2.3 Data Collection Process and Risk of Bias Assessment

Data extraction and assessment of potential risk of bias were also conducted independently and in duplicate by two reviewers. Discrepancies were discussed and, when no consensus was reached, a third reviewer was consulted.

A data extraction form was prepared in Excel format by the authors, pilot‐tested on two studies, and accordingly refined. Extracted data included study context (e.g., country and publication year, number of centers, and sources of funding), study design and duration of follow‐up, population and their demographics, outcomes, prognostic factors (with definitions, including thresholds used for continuous predictors), and measures of association (e.g., odds ratio, risk ratio, and hazard ratio).

The risk of bias in included studies was assessed using the Quality in Prognosis Studies (QUIPS) tool for prognostic factor studies [13]. When any precise definition of some of the study features that were considered in the risk of bias assessment was needed, such as the confounding factors that the studies should account for, experts in the field were contacted and definitions were agreed upon with them.

### 2.4 Assessment of Publication Bias

Publication bias was examined by visual inspection of funnel plots, and by computing the Egger test with the significance level set at 0.05 if 10 or more studies were included in a meta‐analysis. These analyses were performed using the metafunnel and metabias commands in STATA version 17 (StataCorp LLC, College Station, Texas, USA) [14], respectively.

### 2.5 Data Analysis and Synthesis

When possible, the pooled effects of the considered prognostic factors (OS, DFS, PFS, EFS, CSS, and RS) that were reported by more than one study were calculated. Only information coming from multivariate analysis was considered for meta‐analysis. The effect measures (HR or OR) and the corresponding 95% CI were pooled with an indirect variance estimation in meta‐analyses using the Review Manager software (version 5.4.1.), and the results were displayed in forest plots. Results from RCTs and n‐RCTs were combined for factors where the comparison between randomized groups was not the subject of interest and the RCT sample was taken as a whole. Heterogeneity was assessed using the *I* ^2^ statistic. When there was neither clinical nor statistical heterogeneity, a fixed‐effect model was used for analysis. When heterogeneity was present (*I* ^2^ ≥ 50% or *p* < 0.1), a random‐effects model was used for analysis. Potential sources of clinical heterogeneity were anticipated, including BL subtype, age, race/ethnicity, and follow‐up period. When reported in most meta‐analyzed studies, the effect of these study‐level variables was explored using subgroup analyses. Sensitivity analyses were conducted to assess the robustness of the results, excluding one trial at a time and examining the impact of its removal on the summary treatment effect.

### 2.6 Certainty of Evidence Assessment

An assessment of the certainty of evidence based on the GRADE approach was performed for each of the prognostic factors per outcome. The approach considers the following domains to rate down the certainty of evidence: risk of bias, inconsistency, indirectness, imprecision, and publication bias; and the following domains to rate it up: large effect, dose–response gradient, and plausible confounding [15]. Evidence profiles were developed and rated the overall certainty of evidence as high, moderate, low, or very low, depending on the grading of the individual domains.

## 3 Results

**FIGURE 1:**
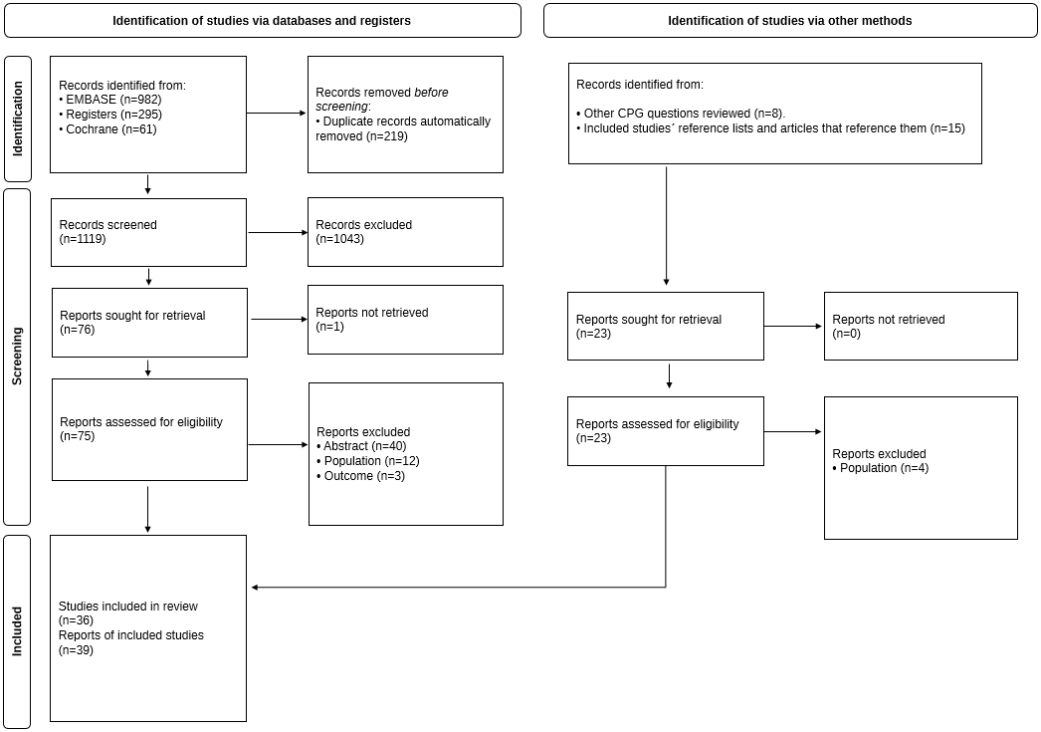
PRISMA flow chart detailing the screening process.

The results of the literature search and study selection processes are shown in Figure 1. The search identified 1119 references after removing duplicates, of which 76 potentially relevant articles were selected for full‐text assessment. Finally, 36 studies [4, 6, 7, 9, 10, 16, 17, 18, 19, 20, 21, 22, 23, 24, 25, 26, 27, 28, 29, 30, 31, 32, 33, 34, 35, 36, 37, 38, 39, 40, 41, 42, 43, 44, 45, 46] reported in 39 papers [4, 6, 7, 8, 9, 10, 16, 17, 18, 19, 20, 21, 22, 23, 24, 25, 26, 27, 28, 29, 30, 31, 32, 33, 34, 35, 36, 37, 38, 39, 40, 41, 42, 43, 44, 45, 46, 47, 48] were eligible for inclusion according to the pre‐established selection criteria. Four papers reported results from the same study [8, 18, 47, 48] and were considered as a single one [18]. There were also three articles reporting retrospective studies conducted on information extracted from the same database, but with different search dates, which were therefore considered as separate studies [6, 36, 40]. Seventeen of these 36 studies were suitable for inclusion in the quantitative synthesis [6, 7, 9, 10, 16, 18, 19, 26, 27, 28, 30, 34, 36, 37, 39, 40, 46]. The list of studies excluded during the full‐text screening along with the main reason for exclusion is provided in Table S2.

### 3.1 Description of Included Studies

The main characteristics of the included studies are summarized in Table 1. All selected studies were published between 2008 and 2022. Two studies were RCTs [7, 28], five were prospective cohort studies [10, 22, 23, 24, 39], and the remaining ones were retrospective cohort studies. Studies were conducted in the United States (8 studies); Spain (5 studies); China (4 studies); South Korea (3 studies); Germany and Italy (2 studies), and Canada, Denmark, Jordan, France, India, Norway, Saudi Arabia, Singapore, South Africa, Sweden, Turkey, and the United Kingdom (1 study).

**TABLE 1:** Characteristics of included studies.

Study, year (Country)	Study design (*N* arms)	*N*	Age	Population	Prognostic factor	Outcomes
Ribrag, 2016 (France) [7]	RCT (2)	257	NR	BL/BLE	Albumin, age, sex, treatment	OS, EFS
Xicoy, 2011 (Germany and Spain) [28]	RCT (2)	75	NR	BL/BLE HIV and non‐HIV	BM, PS	OS, DFS
Hoelzer, 2014 (Germany) [39]	PCS (1)	363	42 (16–85)^c^	BL/BLE	Age, BM, CNS, sex, treatment	OS
Kim, 2021 (South Korea) [10]	PCS (2)	81/60	47 (16–82)^c^/52.5 (18–84)^c^	BL HIV and non‐HIV	Age, PS, risk stratification	OS, PFS
Ribera, 2013 (Spain) [22]	PCS (1)	118	44 (5–83)^c^	BL HIV and non‐HIV	Age, BM, CNS, HIV, PS, sex	OS, DFS
Rizzieri, 2014 (USA) [23]	PCS (1)	105	NR	BL/BLE or BLL	Age	OS, EFS
Roschewski, 2020 (USA) [24]	PCS (1)	113	49 (18–86)^c^	BL HIV and non‐HIV	Age, CNS, risk stratification	OS, EFS
Ahmed, 2011 (Saudi Arabia) [31]	RCS (1)	62	NR	BL HIV and non‐HIV	Age, BM, CNS, HIV, sex	OS, PFS
Albano, 2019 (Italy) [32]	RCS (1)	65	53 (18–80)^a^	BL HIV and non‐HIV	CNS, sex	OS, PFS
Albano, 2019b (Italy) [33]	RCS (1)	61	52 (18–80)^a^	BL HIV and non‐HIV	Age, HIV, sex	OS, PFS
Alderuccio, 2021 (UK)^d^ [47]	RCS (2)	140/109	NR/NR	BL/BLE HIV	CNS, PS	OS, PFS
Barnes, 2011 (USA) [34]	RCS (1)	80	46 (17–80)^c^	BL HIV and non‐HIV	Age, CNS, HIV, risk stratification, treatment	OS, PFS
Castillo, 2013 (USA)^e^ [6]	RCS (1)	2284	49 (20–99)^c^	BL	Age, CNS, sex, race/ethnicity	RS
Chen, 2022 (China) [16]	RCS (2)	229/107	40 (18–70)^c^/NR	SBL	Age, BM, CNS, PS, risk stratification, sex	OS
Chen, 2021 (China) [35]	RCS (1)	123	36 (18–69)^c^	SBL	Risk stratification	OS, EFS, PFS
Choi, 2009 (South Korea) [17]	RCS (1)	38	47 (18–70)^c^	SBL	Age, BM, CNS, PS, sex	OS, PFS
Costa, 2013 (USA)d^e^ [36]	RCS (1)	1922	NR	BL/BLE	Age, sex, race/ethnicity	OS
Evens, 2021 (USA)^d^ [18]	RCS (1)	641	NR	BL HIV and non‐HIV	Age, BM, CNS, PS	OS, PFS
Forero‐Castro, 2015 (Spain) [37]	RCS (1)	16	NR	BL HIV and non‐HIV	Age, BM, CNS, PS, sex, treatment	OS, PFS
Ganesan, 2018 (India) [38]	RCS (1)	32	NR	BL HIV and non‐HIV	Age, HIV, sex, treatment	OS, PFS
Jakobsen, 2020 (Norway & Sweden) [4]	RCS (1)	264	47 (18–81)^c^	SBL	Age, PS	OS, EFS
Jang, 2022 (South Korea) [19]	RCS (1)	64	48 (16–81)^c^	SBL	Age, BM, CNS, PS, sex, treatment	OS, PFS
Ma'koseh, 2021 (Turkey) [20]	RCS (1)	19	33 (19–65)^c^	BL	BM, CNS, treatment	OS, EFS
Malkan, 2016 (Jordan) [21]	RCS (1)	25	39 (16–63)^c^	BLE	Treatment	OS, DFS
Mukhtar, 2017 (USA)^e^ [40]	RCS (2)	1994/757	53.6 ± 8.8/78.5 ± 4.8^b^	BL/BLE	BM, CNS, race/ethnicity	RS
Musekwa, 2020 (South Africa) [41]	RCS (1)	50	37 (31–43)^c^	BL HIV and non‐HIV	HIV, treatment	OS
Olszewski, 2021 (Denmark)^d^ [8]	RCS (2)	633/457	47 (33–59)/46 (34–59)^c^	BL HIV and non‐HIV	Age, CNS, PS	OS, PFS
Oriol, 2008 (Spain) [42]	RCS (1)	36	NR	BL HIV and non‐HIV	HIV	OS, DFS
Phillips, 2020 (UK) [43]	RCS (1)	27	NR	BL HIV and non‐HIV	Treatment	OS, DFS
Sakarou, 2019 (Germany) [44]	RCS (1)	29	NR	BL HIV and non‐HIV	Age, treatment	OS, DFS
Tan, 2022 (Singapore) [45]	RCS (1)	34	48 (41–57)^c^	BL/BLE HIV and non‐HIV	HIV	OS, PFS
Wang, 2021 (China) [26]	RCS (1)	78	46 (35–60)^c^	BL HIV	Albumin, treatment	OS
Wang, 2015 (China) [25]	RCS (1)	62	38 (18–75)^c^	SBL	Age, BM, PS, sex, risk stratification	OS, PFS
Wästerlid, 2013 (Denmark & Sweden) [46]	RCS (1)	258	56 (15–93)^c^	BL/BLE	Age, CNS, PS, treatment	OS
Wästerlid, 2011 (Sweden) [9]	RCS (1)	156	56 (16–93)^c^	BL	Age, CNS, PS, treatment	OS
Wildes, 2014 (USA) [27]	RCS (1)	35	44 (20–74)^c^	BL/BLE HIV	Age, BM, CNS, PS, sex, treatment	OS, EFS
Xicoy, 2014 (Germany and Spain) [29]	RCS (1)	81	43 (20–69)^c^	BL/BLE HIV	BM, PS	OS, DFS, EFS, PFS
Zayac, 2021 (USA)^d^ [48]	RCS (1)	641	NR	BL HIV and non‐HIV	BM, CNS, HIV, PS	OS, PFS
Zhu, 2018 (Canada) [30]	RCS (1)	81	47 (18–72)^c^	BL HIV and non‐HIV	Age, BM, HIV, treatment	OS, PFS

Abbreviations: BM: bone marrow involvement; BL: Burkitt's lymphoma; BLE: Burkitt's leukemia; BLL: Burkitt‐like lymphoma; CNS: central nervous system involvement; DFS: disease‐free survival; EFS: event‐free survival; HIV: human immunodeficiency virus; *N*: sample size; NR: not reported; NA: not applicable; OS: overall survival; PCS: prospective cohort study; PFS: progresion‐free survival; PS: performance status; RCS: retrospective cohort study; RS: relative survival; SBL: sporadic Burkitt's lymphoma; UK: United Kingdom; USA: United States of America.

^a^ Mean (Range).

^b^ Mean ± SD.

^c^ Median (IQR).

^d^ Publications reporting analyses derived from the same sample.

^e^ Publications reporting analyses derived from the same database.

Across the 36 studies, 10,882 BL patients were recruited, of which 599 had HIV. The largest study recruited 2751 patients [40], whereas the smallest one only had 16 patients [37]. The median age of the patients was 39.4 years, ranging from 15 to 93. Six studies focused specifically on patients with sporadic BL [4, 16, 17, 19, 25, 35], and three on HIV‐related BL patients [26, 27, 29]. Additionally, 27 studies examined patients with mixed subtypes of BL [6, 7, 9, 10, 18, 20, 21, 22, 23, 24, 28, 30, 31, 32, 33, 34, 36, 37, 38, 39, 40, 41, 42, 43, 44, 45, 46].

Of the 36 studies included, 34 reported on OS [4, 7, 9, 10, 16, 17, 18, 19, 20, 21, 22, 23, 24, 25, 26, 27, 28, 29, 30, 31, 32, 33, 34, 35, 36, 37, 38, 39, 41, 42, 43, 44, 45, 46], 15 reported on PFS [10, 17, 18, 19, 25, 29, 30, 31, 32, 33, 34, 35, 37, 38, 45], seven reported on DFS [21, 22, 28, 29, 42, 43, 44], eight on EFS, [4, 7, 20, 23, 24, 27, 29, 35] and two on RS [6, 40]. Study follow‐up periods ranged from 2 to 13 years.

### 3.2 Risk‐of‐Bias in Included Studies

Out of the 36 included studies, eight presented an overall low risk of bias [7, 18, 22, 25, 26, 30, 38, 46]; four were rated as having an overall medium risk of bias, as two domains were assessed as being medium risk [16, 17, 29, 39]; and the remaining studies had a high overall risk of bias due to at least one domain or item being rated as high risk or three or more domains rated as medium risk of bias [4, 6, 9, 10, 19, 20, 21, 23, 24, 27, 28, 31, 32, 33, 34, 35, 36, 37, 40, 41, 42, 43, 44, 45]. Studies were rated as medium or high risk of bias mainly due to the fact they did not account for some important confounders, because the statistical methods applied were not detailed enough to ensure an adequate assessment, the strategy for model building was not appropriate for the study objectives and/or they did not have a previously published study protocol. Table S3 provides detailed judgments for each of the assessed risk‐of‐bias domains.

### 3.3 Prognostic Factor for Survival in BL Patients

Twelve candidate prognostic factors for survival were assessed from the 36 studies considered. The results of all meta‐analyses can be found in Table S4. Table S5 provides the evidence profiles for all considered prognostic factors.

No evidence of publication bias was detected through visual assessment (Figure S1.1–1.14), except for potential risk in the association of age with OS and PFS (Figure S1.1 and 1.2), and the association of performance status with OS (Figure S1.9). It was not possible to perform the Egger test for any of the outcomes due to the small number of studies included in each MA.

### 3.4 Age

**FIGURE 2:**
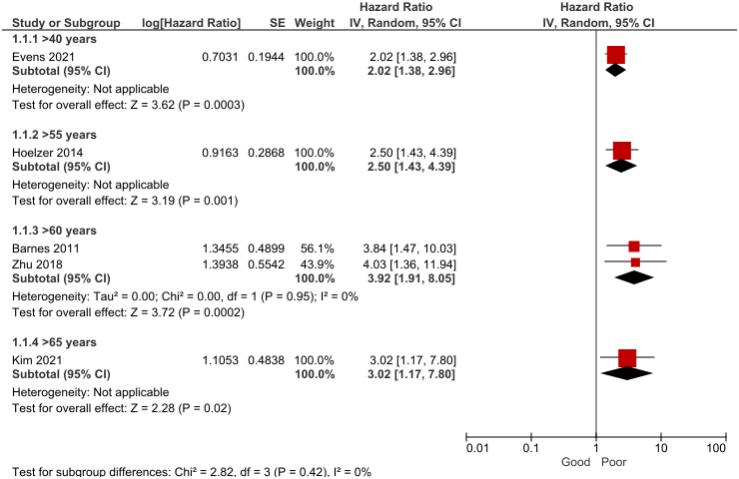
Forest plot for the association between age and overall survival at 3–5 years.

**FIGURE 3:**
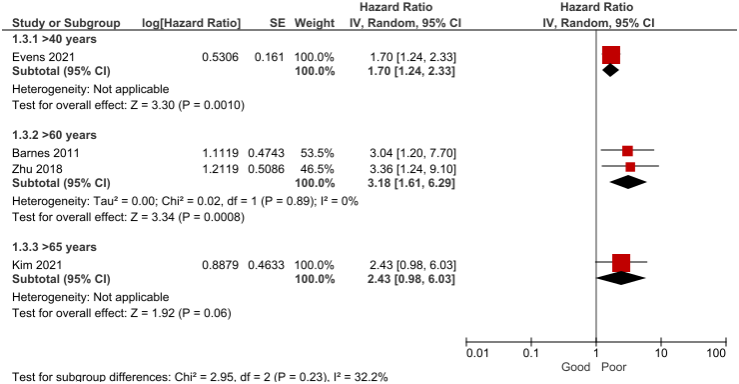
Forest plot for the association between age and progression‐free survival at 3–5 years.

Older age was found to be associated with poorer OS (Figure 2) [10, 18, 30, 34, 39] and shorter PFS (Figure 3) [10, 18, 30, 34] at 3–5 years. The certainty of the evidence was rated as moderate for both association estimates.

### 3.5 Sex

There were no significant differences between men and women with BL in OS [36, 39] (Figure S2.1) or RS (fixed effects, HR = 1.15, 95% CI: 0.99, 1.34, *p* = 0.07, *k* = 1, *N* = 2284) [6] at 5 years. The certainty of evidence was rated as very low for OS and as low for RS.

### 3.6 Race/Ethnicity

Black patients with BL exhibited a significantly poorer OS (fixed effects, HR = 1.28, 95% CI 1.05, 1.56, *p* = 0.01, *k* = 1, *N* = 1749) [36] and RS (fixed effects, HR = 1.60, 95% CI 1.30, 1.97, *p* < 0.00001, *k* = 1, *N* = 1695) [6] at 5 years compared to those who were white. The certainty of evidence was rated as moderate for OS and as low for RS. No significant difference in RS was found in Hispanic patients (fixed effects, HR = 1.08, 95% CI 0.90, 1.30, *p* = 0.41, *k* = 1) [6] or Asian‐Pacific patients (fixed effects, HR = 0.92, 95% CI 0.73, 1.16, *p* = 0.49, *k* = 1) [40] compared to white patients at 5 years. The certainty of evidence for both outcomes was rated as low. Data for other races/ethnicities could not be meta‐analyzed due to heterogeneous group definitions.

### 3.7 HIV

There were no significant differences in OS within a period of 3–5 years when comparing BL patients with HIV and those without HIV (fixed effects, HR = 1.53, 95% CI 0.55, 4.27, *p* = 0.42, *k* = 2) [30, 34]. The subgroup analysis showed that the absence of significant differences remained with the independence of the follow‐up period, at 3 years (fixed effects, HR = 1.58, 95% CI 0.50, 4.99, *p* = 0.44, *k* = 1) [34] and at 5 years (fixed effects, HR = 1.35, 95% CI 0.14, 13.02, *p* = 0.80, *k* = 1) [30].

There was also no significant association in PFS at 5 years between BL patients with HIV and those without HIV (fixed effects, HR = 1.17, 95% CI: 0.38, 3.60, *p* = 0.78, *k* = 1, *N* = 80) [34]. The certainty of evidence was rated as low for both outcomes.

### 3.8 Performance Status

Performance status was assessed with the Eastern Cooperative Oncology Group (ECOG) performance status scale (ECOG‐PS) [49]. This scale measures how cancer impacts a patient's daily living abilities and offers a rating from 0 (fully active and able) to 5 (dead).

**FIGURE 4:**
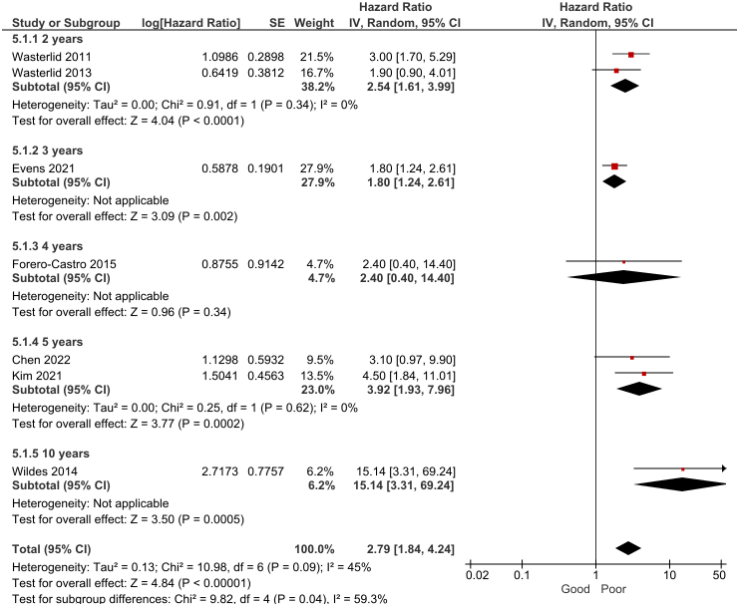
Forest plot for the association between performance status (ECOG‐PS grade ≥ 2) and OS at 2–10 years.

BL patients with ECOG‐PS grade ≥ 2 (capable of self‐care but unable to carry out any work activities) had significantly poorer OS than participants with ECOG‐PS grade < 2 during a 2–10 year follow‐up period [9, 10, 16, 18, 27, 37, 46] (Figure 4). The certainty of evidence was assessed as low for this outcome. In the subgroup analysis by follow‐up time, this difference was found at 2 years [9, 46], 3 years [18], 5 years [10, 16], and 10 years [27] but no significant association was found at 4 years [37].

The sensitivity analysis showed heterogeneity in the overall effect was caused by one study alone [27], when removed from the meta‐analysis, no significant heterogeneity or subgroup differences were observed and the pooled effect remained robust (random effects, HR = 2.29, 95% CI: 1.74, 3.01, *p* < 0.00001, *k* = 6, *N* = 1382).

**FIGURE 5:**
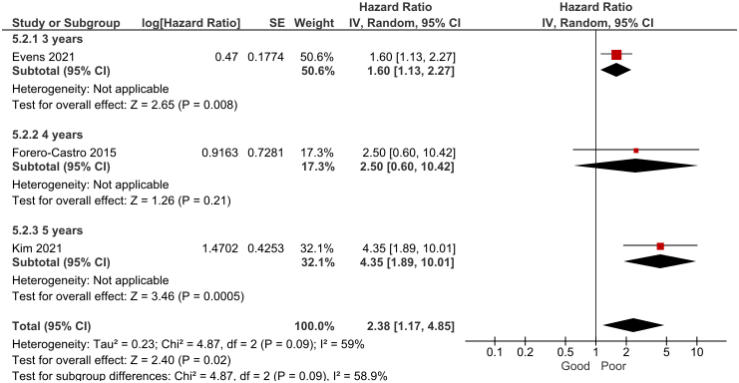
Forest plot for the association between performance status (ECOG‐PS grade ≥ 2) and PFS at 3–5 years.

The analysis revealed that BL patients with ECOG‐PS grade ≥ 2 also had significantly poorer PFS (Figure 5). The certainty of evidence was assessed as being low for this outcome. In the subgroup analysis by follow‐up time, this effect was found at 3 years [18] and 5 years [10], but no significant association was observed at 4 years [37].

### 3.9 Risk Stratification

The authors identified low‐certainty evidence that there is no significant difference in OS over a period of 3 years (fixed effects, HR = 3.81, 95% CI 0.85, 17.08, *p* = 0.08, *k* = 1, *N* = 81) [34] between low‐risk and high‐risk BL patients.

### 3.10 BM Involvement

**FIGURE 6:**
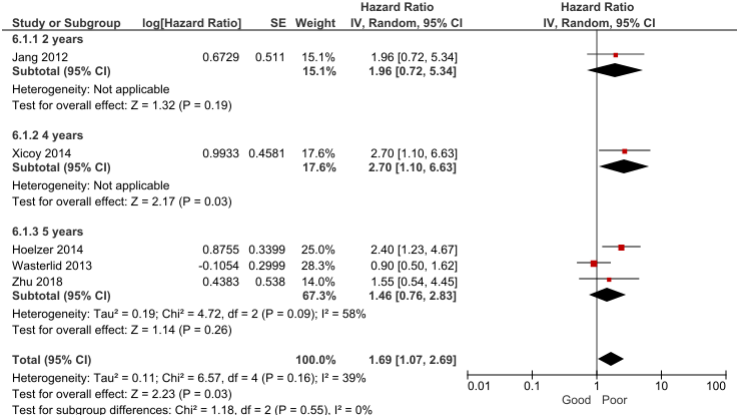
Forest plot for the association between BM involvement and OS at 2–5 years.

Patients with BM involvement presented significantly poorer OS when compared to those without BM involvement [19, 29, 30, 39, 46] (Figure 6). In the subgroup analysis by follow‐up time, the effect was significant at 4 years [29], but no significant impact of BM involvement in OS was found at 2 years [19] and 5 years [30, 39, 46]. The certainty of evidence was assessed as being moderate for OS.

No association was found between BM involvement and PFS at 4–5 years [29, 30] (Figure S2.2). In the subgroup analysis by follow‐up time, the effect remained not significant at 4 years [29] and at 5 years [30]. The certainty of evidence was rated as low for PFS.

No association was found either between BM involvement and RS at 5 years (fixed effects, HR = 1.25, 95% CI: 0.99, 1.59, *p* = 0.45, *k* = 1, *N* = 2751) [40]. The certainty of this evidence was rated as low.

### 3.11 CNS Involvement

**FIGURE 7:**
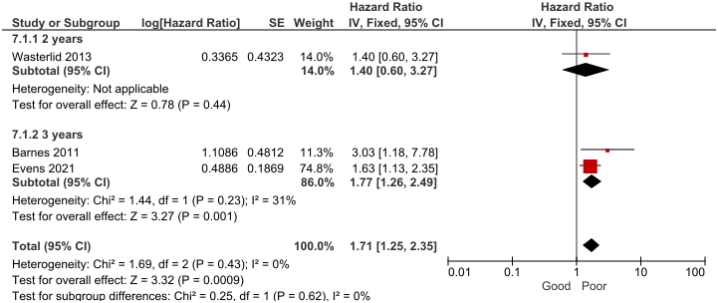
Forest plot for the association between CNS involvement and OS at 2–3 years.

**FIGURE 8:**
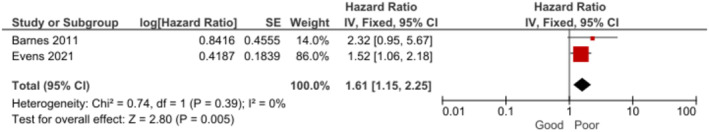
Forest plot for the association between CNS involvement and PFS at 3 years.

Patients with CNS involvement had significantly poorer OS [18, 34, 46] at 2–3 years (Figure 7) and PFS at 3 years (Figure 8) [18, 34] than patients without CNS involvement. In the subgroup analysis, no association between CNS involvement and OS was found at specifically 2 years [46]. The certainty of evidence for both outcomes was rated as high.

### 3.12 Albumin Levels

**FIGURE 9:**
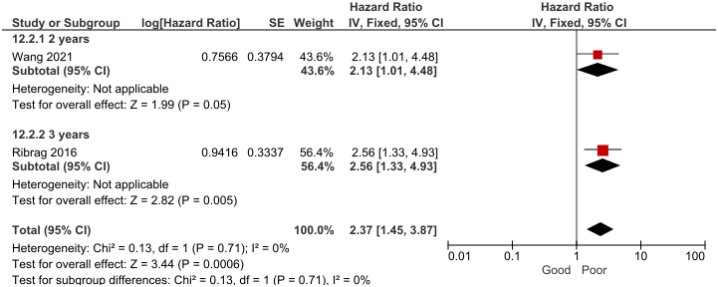
Forest plot for the association between albumin levels < 30 and 35 g/L and OS at 2–3 years.

Patients with albumin levels < 30–35 g/L had significantly lower OS at 2–3 years when compared to those with ≥ 30–35 g/L albumin levels [7, 26] (Figure 9). In the subgroup analysis by follow‐up time, the effect remained significant both at 2 and 3 years. The certainty of this evidence was rated as high.

### 3.13 Treatment With Rituximab

**FIGURE 10:**
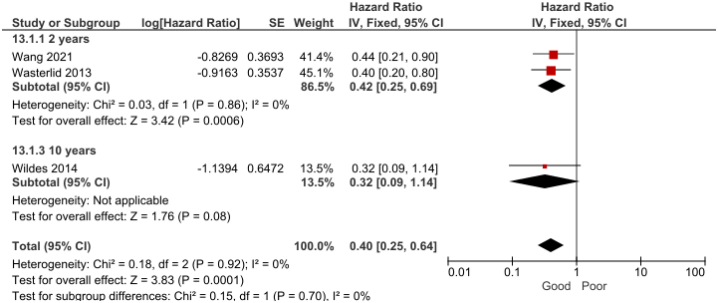
Forest plot for the association between treatment with rituximab and OS at 2–10 years.

Patients who received treatment with rituximab presented a significantly better OS at 2–10 years when compared to those who did not receive rituximab [26, 27, 46] (Figure 10). OS at 2 years [26, 46] was found to be significant during the subgroup analysis by follow‐up time. However, no significant association was found between rituximab treatment and OS at 10 years [27]. The certainty of evidence for OS was rated as high.

No significant association was found between treatment with rituximab and PFS at 3–5 years (random effects, HR = 0.26, 95% CI: 0.04 to 1.60, *p* = 0.15, *k* = 2, *N* = 161; *I* ^2^ = 78%). In the subgroup analysis by follow‐up period, no association was found at 3 years (fixed effects, HR = 0.59, 95% CI: 0.26–1.34, *p* = 0.21, *k* = 1, *N* = 80) [34]. Nevertheless, BL patients who received treatment with rituximab presented significantly better PFS at 5 years (fixed effects, HR = 0.09, 95% CI: 0.02, 0.41, *p* = 0.002, *k* = 1, *N* = 81) [30]. The certainty of evidence for PFS was rated as very low.

### 3.14 Treatment With Methotrexate

Patients who received treatment with methotrexate presented significantly better OS (fixed effects, HR = 0.28, 95% CI: 0.09, 0.87, *p* = 0.03, *k* = 1, *N* = 81) [30], and PFS (fixed effects, HR = 0.28, 95% CI: 0.10, 0.78, *p* = 0.02, *k* = 1, *N* = 81) [30] at 3 years when compared with those who did not receive methotrexate. The certainty of evidence was rated as high for both OS and PFS.

### 3.15 Transplantation and Comorbidities

It was not possible to synthesize the relationship between transplants or comorbidities and the outcome measures considered with the data reported in the included studies.

## 4 Discussion

The authors conducted a comprehensive systematic review of the literature to assess 12 potential prognostic factors for survival in patients with BL. Several significant associations were observed. Older age was found to be associated with poorer OS and shorter PFS. Black patients presented significantly lower OS and RS. Patients with higher performance status (ECOG‐PS score ≥ 2) had significantly poorer OS and PFS. Bone marrow involvement was associated with poorer OS, but no significant association was found with PFS. Patients with CNS involvement had significantly poorer OS and PFS. Lower albumin levels (< 30–35 g/L) were associated with lower OS. Treatment with rituximab and treatment with methotrexate were significantly associated with better OS and PFS. On the other hand, no significant differences in OS were found between men and women. There were no significant associations between HIV status and OS or PFS, and risk stratification did not show any significant associations with OS.

In our analysis, age, performance status ≥ 2, and CNS involvement, which all are components of the BL International Prognostic Index (BL‐IPI) [8], showed a statistically significant impact on survival in patients with BL. Therefore, the BL‐IPI scoring system may represent an interesting model to test in prospective clinical trials with large cohorts of patients and could help to tailor new stratifications and modulate treatment intensity.

The identification and assessment of robust prognostic factors can inform treatment decisions and improve patient care aiding the determination of optimal therapies for selected subgroups of patients. In this sense, it may help to reduce treatment density in very good prognostic patients (without bone marrow infiltration, no CNS involvement, and localized disease), but also to identify very poor prognosis patients who need new therapies (such as bispecific antibodies or CAR‐T cell treatment).

Recognizing these prognostic factors also highlights the need for personalized prognostic models to guide the clinical management of BL and underscores the need for further research in this area. Recent prospective studies have used prognostic factors for patient stratification and treatment intensity modulation [7, 22, 23, 24, 39, 43, 50, 51]. This use of predictive factors was not identical in the different recently reported studies. Unfortunately, the choice was not uniform, which increases the complexity of a direct comparison of the different studies. The most common factors used for treatment stratification among these studies were age, International Prognostic Index, bone marrow involvement, and CNS localization. When therapy is adapted following these factors and the treatment modulation is efficient, they may lose their prognostic significance. One example is CNS infiltration and treatment in the LMB trial where very high doses of methotrexate and cytarabine are used in the group of patients with CNS involvement [7]. In this case, the prognostic value of CNS infiltration was no longer significant in the multivariate analysis, suggesting the effectiveness of treatment stratification.

The present review benefits from several methodological strengths and rigorous procedures, including a broad literature search, an independent screening performed in duplicate, a thorough data extraction process, and an assessment of the certainty of evidence performed on the basis of a structured and explicit framework. Furthermore, a quantitative synthesis of the results was performed, investigating important sources of heterogeneity. To the best of the authors' knowledge, this review is the first and most comprehensive evidence review on prognostic factors in adult patients with BL to date.

The main limitation of the present review is that the available evidence comes primarily from retrospective studies, a study design where poor control over the exposure factor, covariates, and potential confounders and bias are common traits. In addition, a further potential limitation of this review lies in the possibility that some studies have not been included, either because they are not written in English or because they are not indexed in the consulted electronic databases. Lastly, included studies often measured the prognostic effect using different cut‐off points for each factor and diverse time‐frames for survival outcomes, which further hindered direct comparison between studies. These limitations could difficult the generalization of certain findings, particularly those related to HIV‐positive patients. Some of the studies included in this analysis did not provide sufficient information on this population, which could have affected the accuracy of the results. Consequently, it is important to consider this fact when interpreting the findings while emphasizing the need for more specific and comprehensive studies on prognostic factors in BL for the HIV‐positive population.

## 5 Conclusion

This work systematically identified and assessed several prognostic factors for survival in BL patients, offering a step forward in the management of this aggressive disease. Future research, particularly prospective studies, is essential to confirm these findings and to explore new prognostic indicators, ultimately aiming to improve patient outcomes through more informed and individualized therapeutic strategies.

## Author Contributions

**Aythami de Armas‐Castellano:** conceptualization (supporting), data curation (equal), formal analysis (equal), investigation (equal), methodology (equal), visualization (lead), writing – original draft (lead), writing – review and editing (lead). **Diego Infante‐Ventura:** conceptualization (supporting), data curation (equal), formal analysis (equal), investigation (equal), methodology (equal), visualization (equal), writing – original draft (equal), writing – review and editing (supporting). **Tasmania del Pino‐Sedeño:** conceptualization (equal), data curation (equal), formal analysis (equal), investigation (equal), methodology (lead), supervision (equal), visualization (supporting), writing – original draft (supporting), writing – review and editing (supporting). **Yadira González Hernández:** conceptualization (supporting), data curation (supporting), formal analysis (supporting), investigation (supporting), writing – review and editing (supporting). **Raul Quiros:** validation (supporting), writing – original draft (supporting), writing – review and editing (supporting). **Beatriz León‐Salas:** conceptualization (supporting), data curation (supporting), investigation (supporting), methodology (supporting), writing – review and editing (supporting). **Vincent Ribrag:** conceptualization (supporting), data curation (supporting), investigation (supporting), validation (lead), writing – original draft (supporting), writing – review and editing (supporting). **María M. Trujillo‐Martín:** conceptualization (lead), investigation (supporting), methodology (supporting), project administration (lead), supervision (lead), writing – review and editing (supporting). **EuroBlood working group ERN:** conceptualization (supporting), data curation (supporting), investigation (supporting), project administration (supporting), supervision (supporting), validation (supporting).

## Ethics Statement

The authors have nothing to report.

## Conflicts of Interest

The authors declare no conflicts of interest.

## Supporting information


Figure S1.

Figure S2.

Table S1.

Table S2.

Table S3.

Table S4.

Table S5.

## Data Availability

Data sharing is not applicable to this article as no datasets were generated or analyzed during the current study.
