# Supplementary material for: Prognostic Factors for Survival in Adults With Burkitt Lymphoma: A Systematic Review
Source: Cancer Med. 2025 Jan 29;14(3):e70513. doi: 10.1002/cam4.70513 (PMC11775923; doi:10.1002/cam4.70513)

Figure S1.1. Funnel plot-publication bias: multivariate analysis of age and overall survival (OS) at 3-5 years.

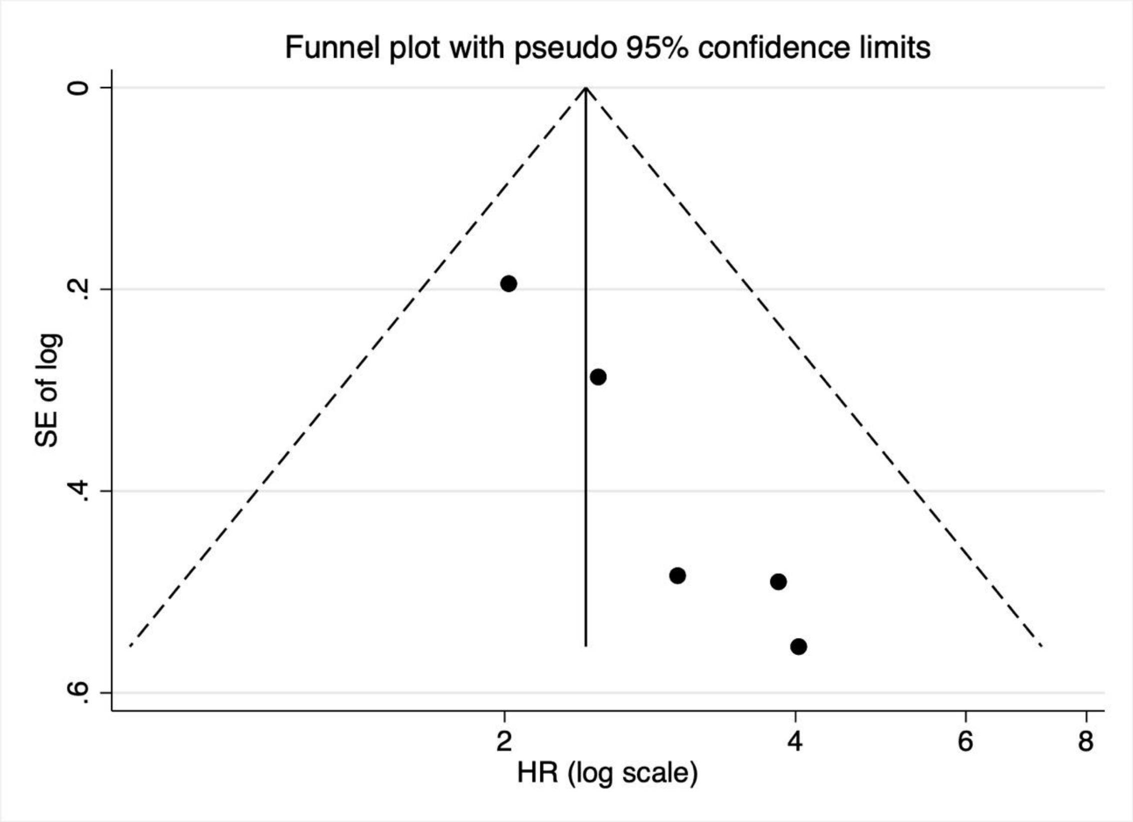

Figure S1.2. Funnel plot-publication bias: multivariate analysis of age and progression-free survival (PFS) at 3-5 years.

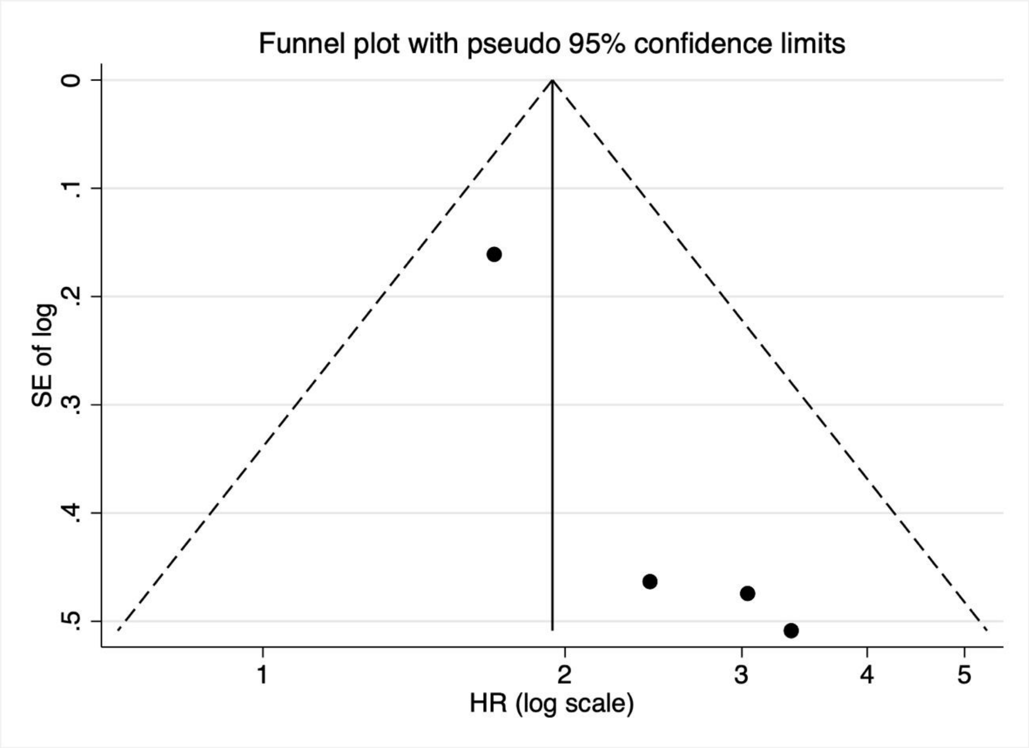

Figure S1.3. Funnel plot-publication bias: multivariate analysis of Albumin and OS at 2-3 years.

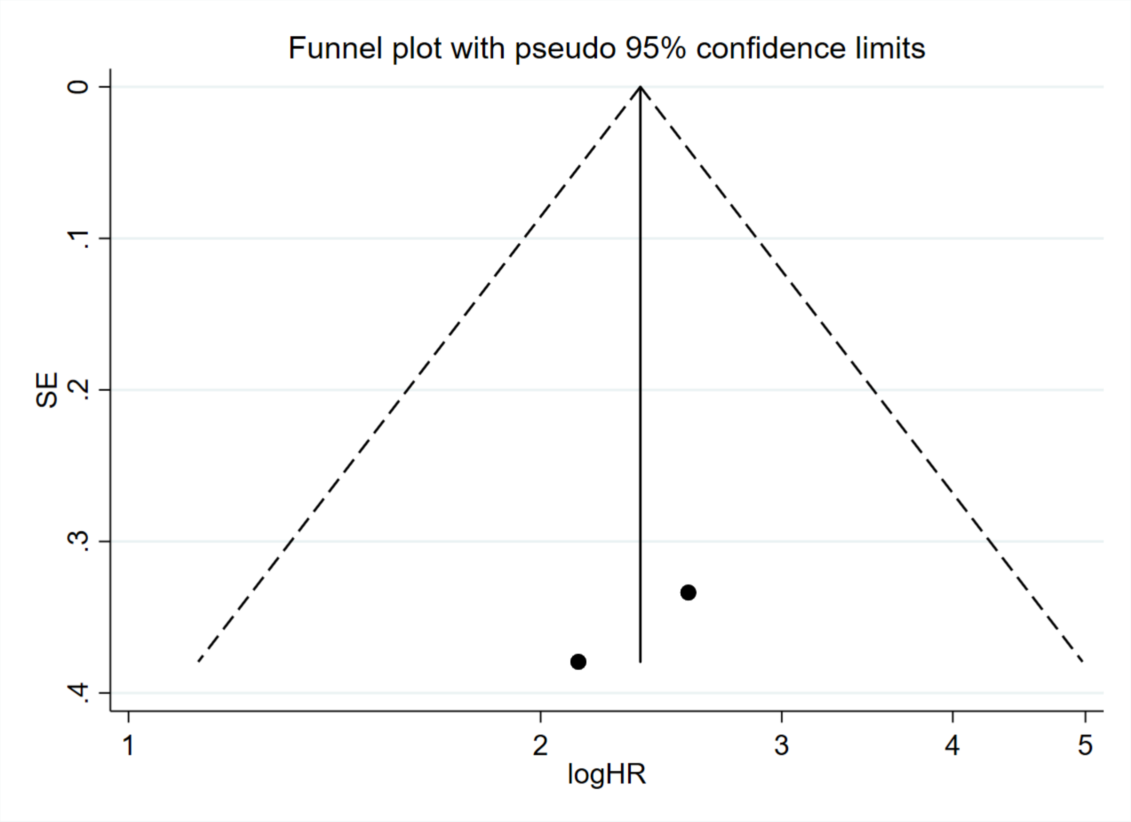

Figure S1.4. Funnel plot-publication bias: multivariate analysis of BM involvement and OS at 2-5 years.

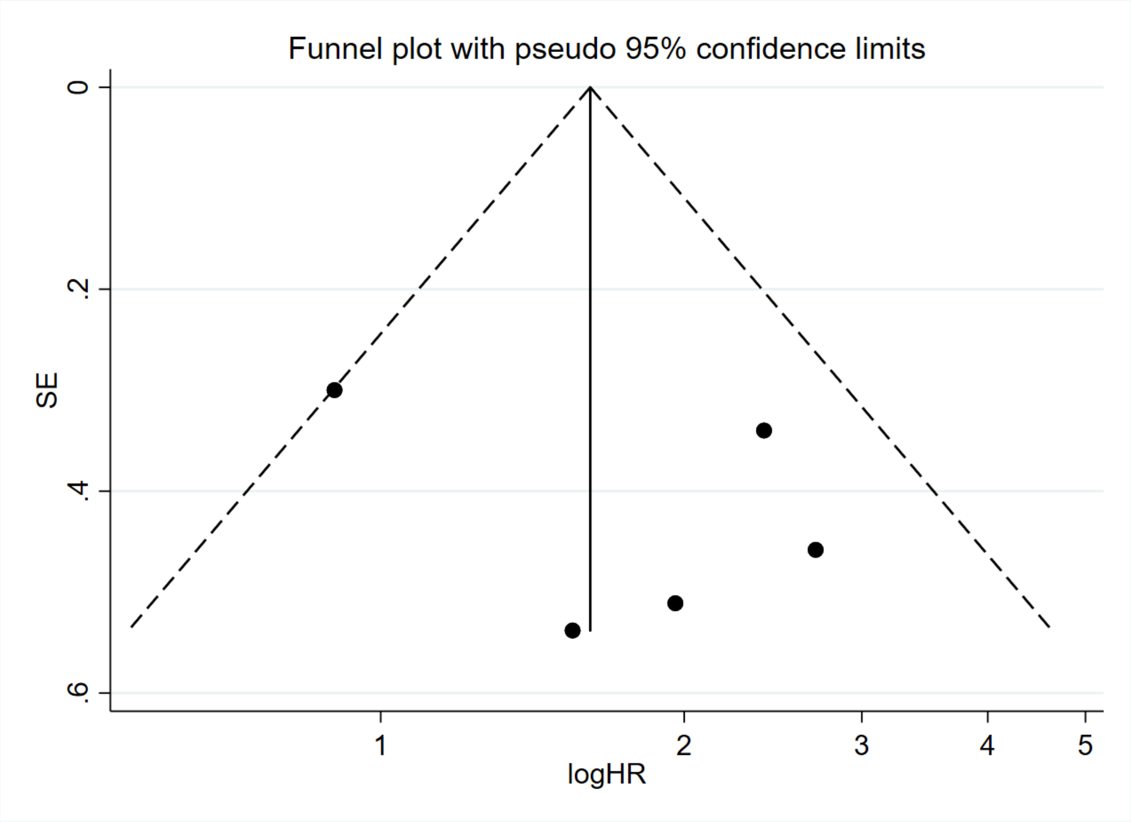

Figure S1.5. Funnel plot-publication bias: multivariate analysis of BM involvement and PFS at 4-5 years.

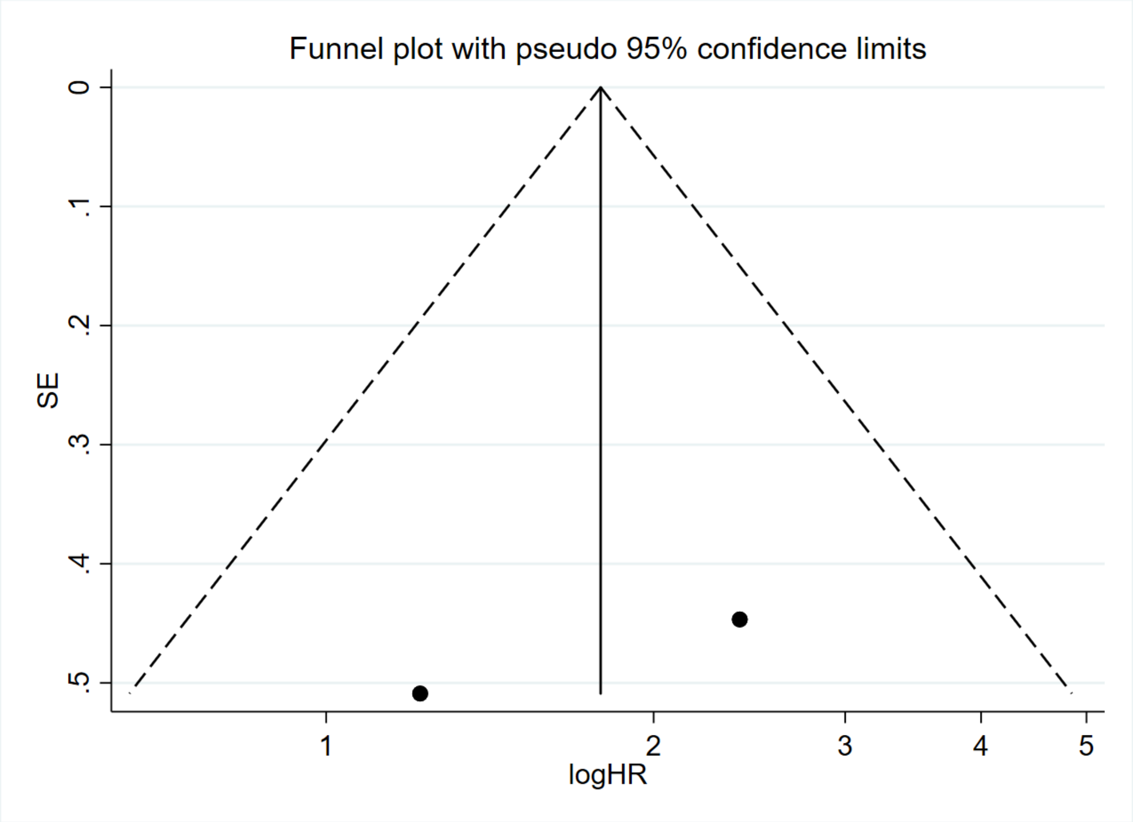

Figure S1.6. Funnel plot-publication bias: multivariate analysis of CNS involvement and OS at 2-3 years.

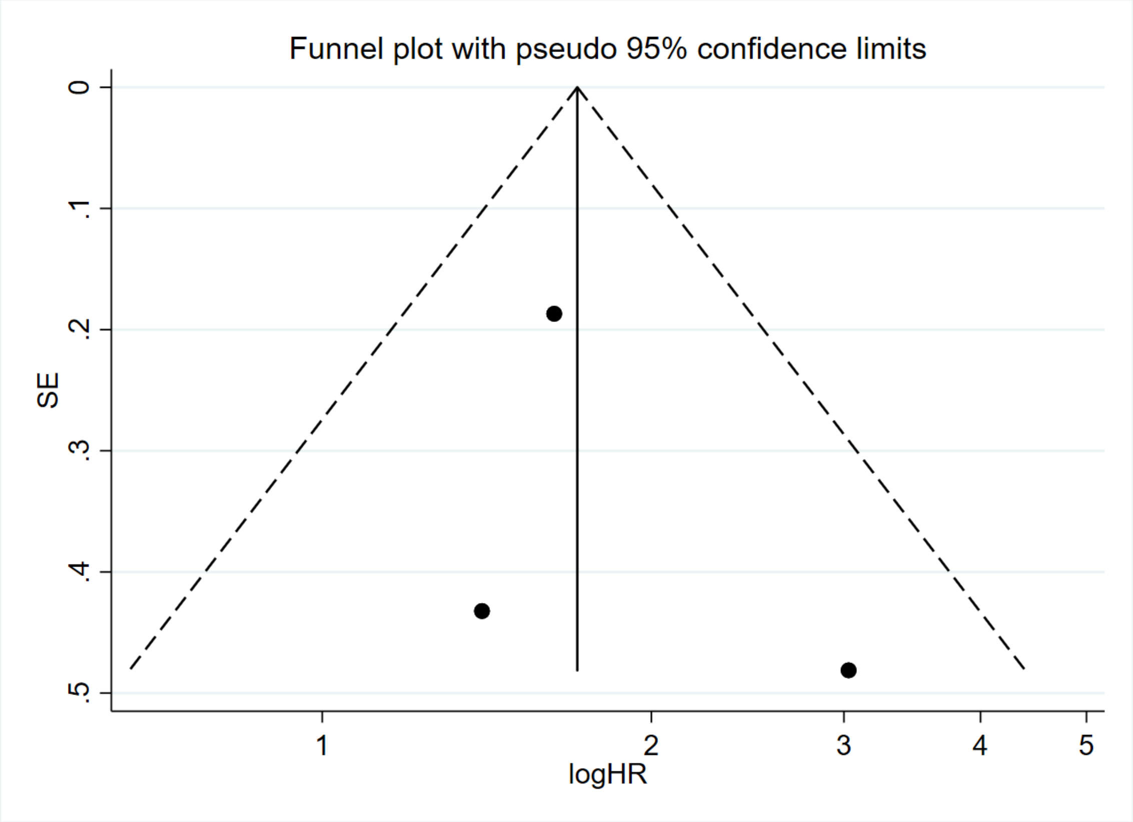

Figure S1.7. Funnel plot-publication bias: multivariate analysis of central nervous system involvement and PFS at 3 years.

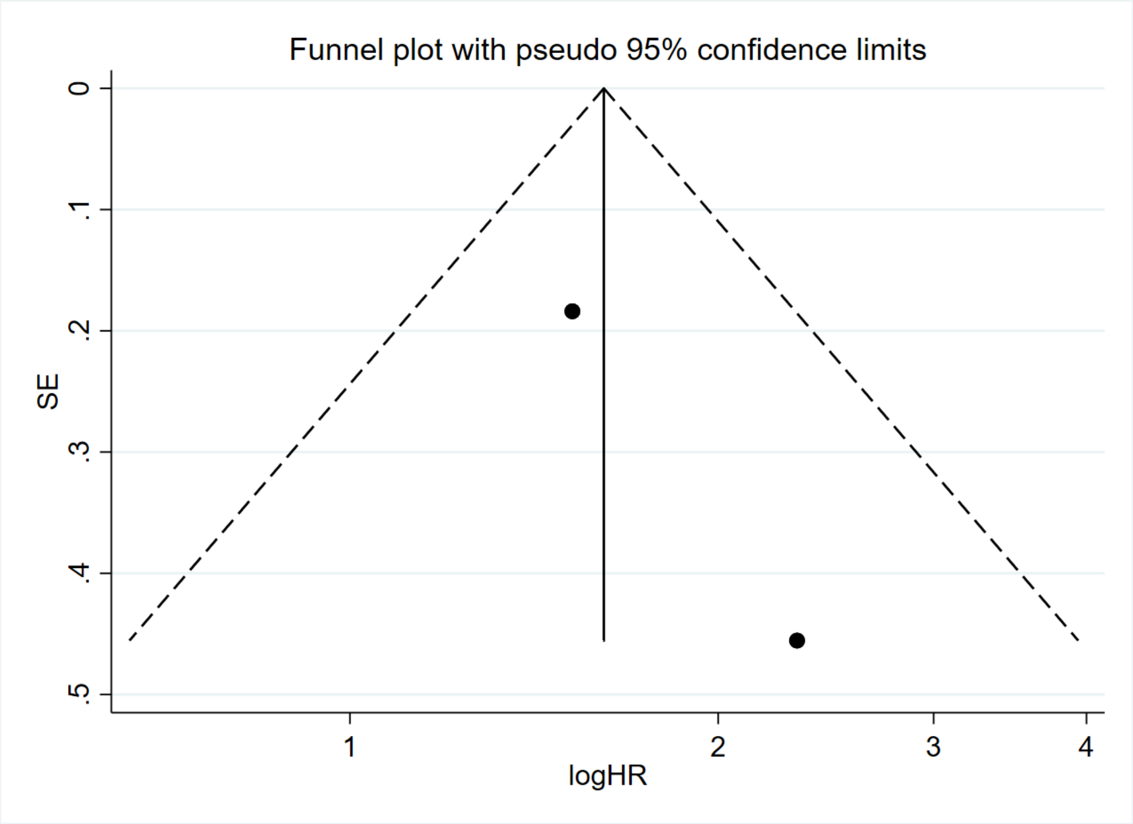

Figure S1.8. Funnel plot-publication bias: multivariate analysis of HIV and OS at 3-5 years.

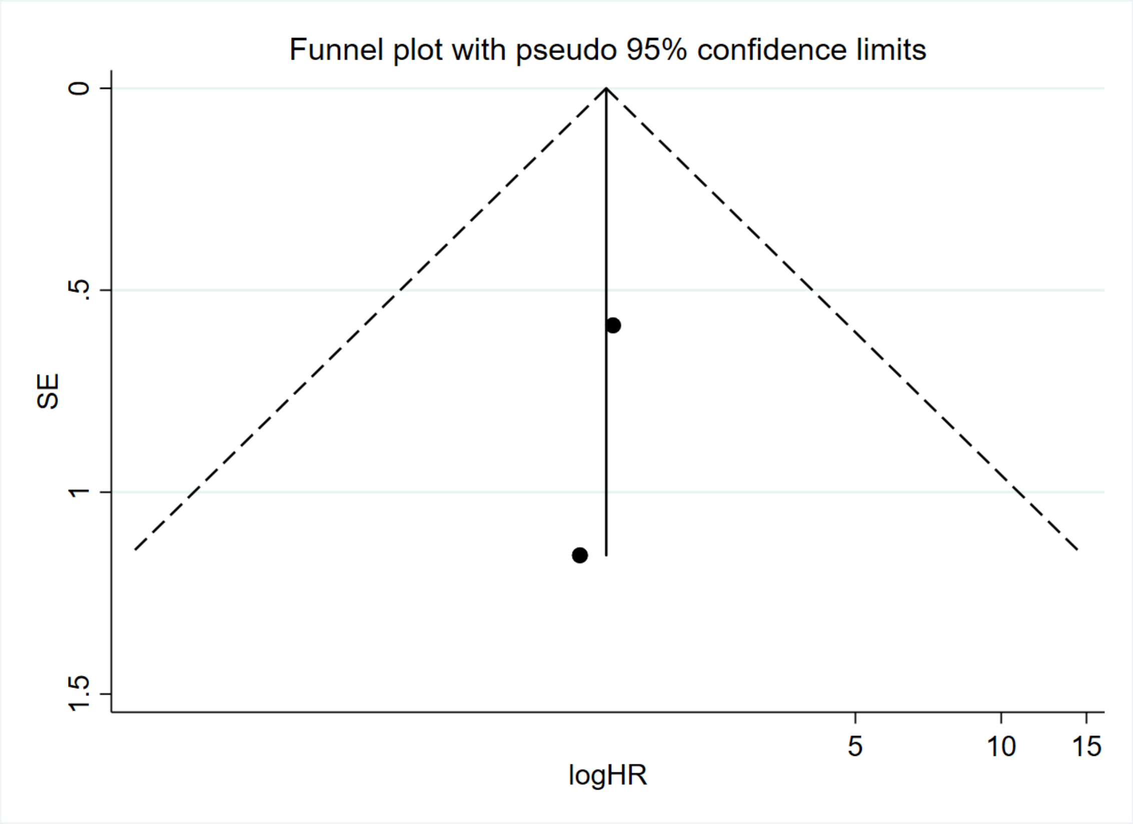

Figure S1.9. Funnel plot-publication bias: multivariate analysis of Performance Status and OS at 2-10 years.

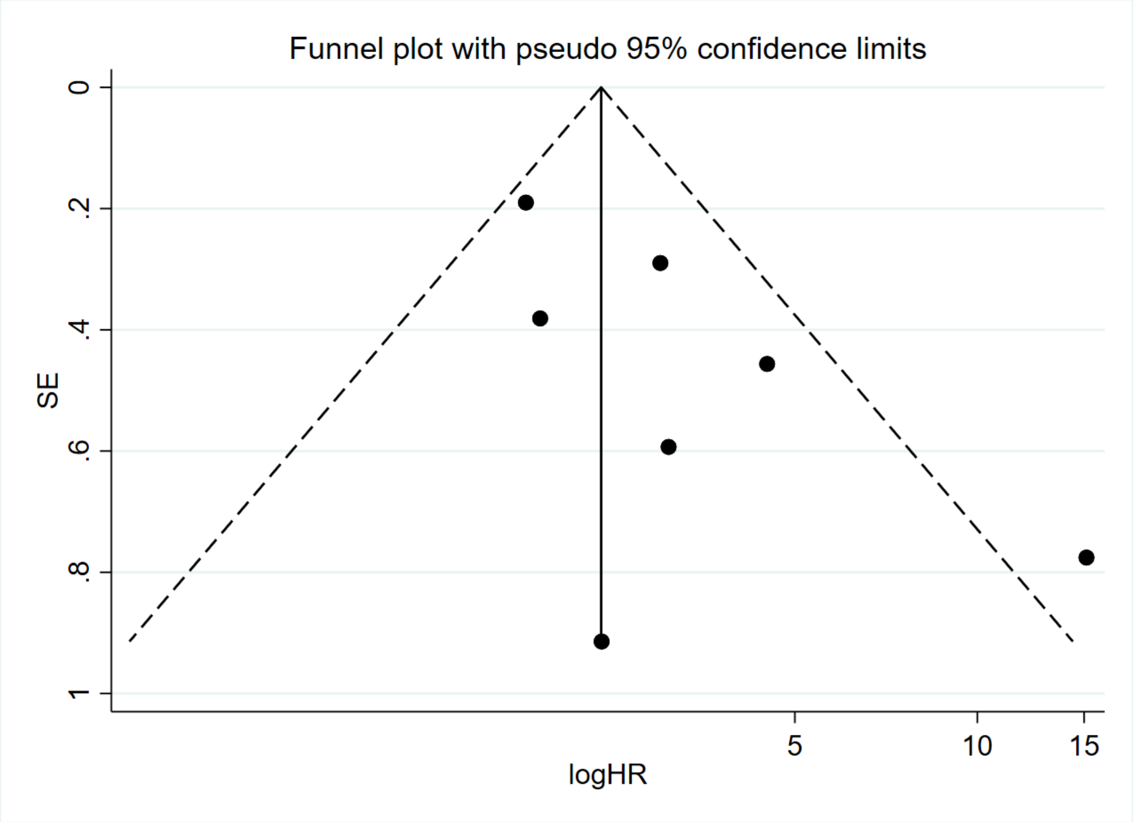

Figure S1.10. Funnel plot-publication bias: multivariate analysis of Performance Status and PFS at 3-5 years.

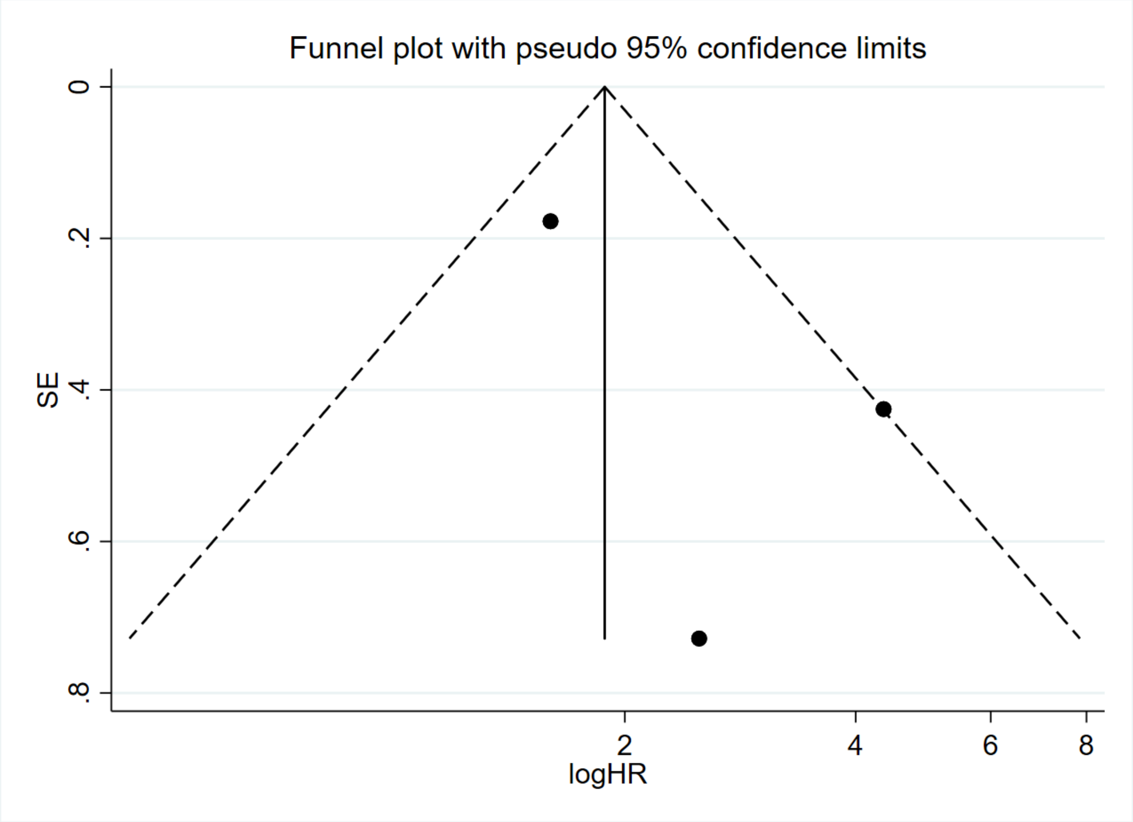

Figure S1.11. Funnel plot-publication bias: multivariate analysis of race and relative survival (RS) at 5 years.

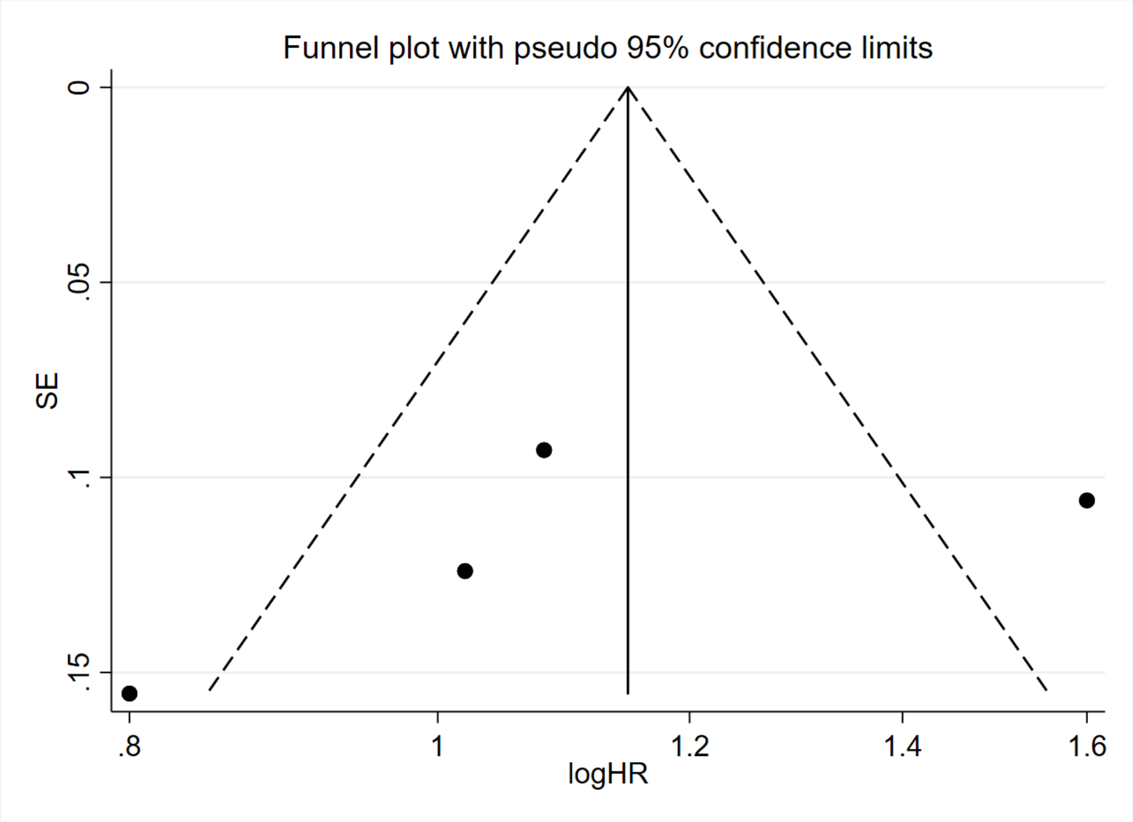

Figure S1.12. Funnel plot-publication bias: multivariate analysis of sex and OS at 5 years.

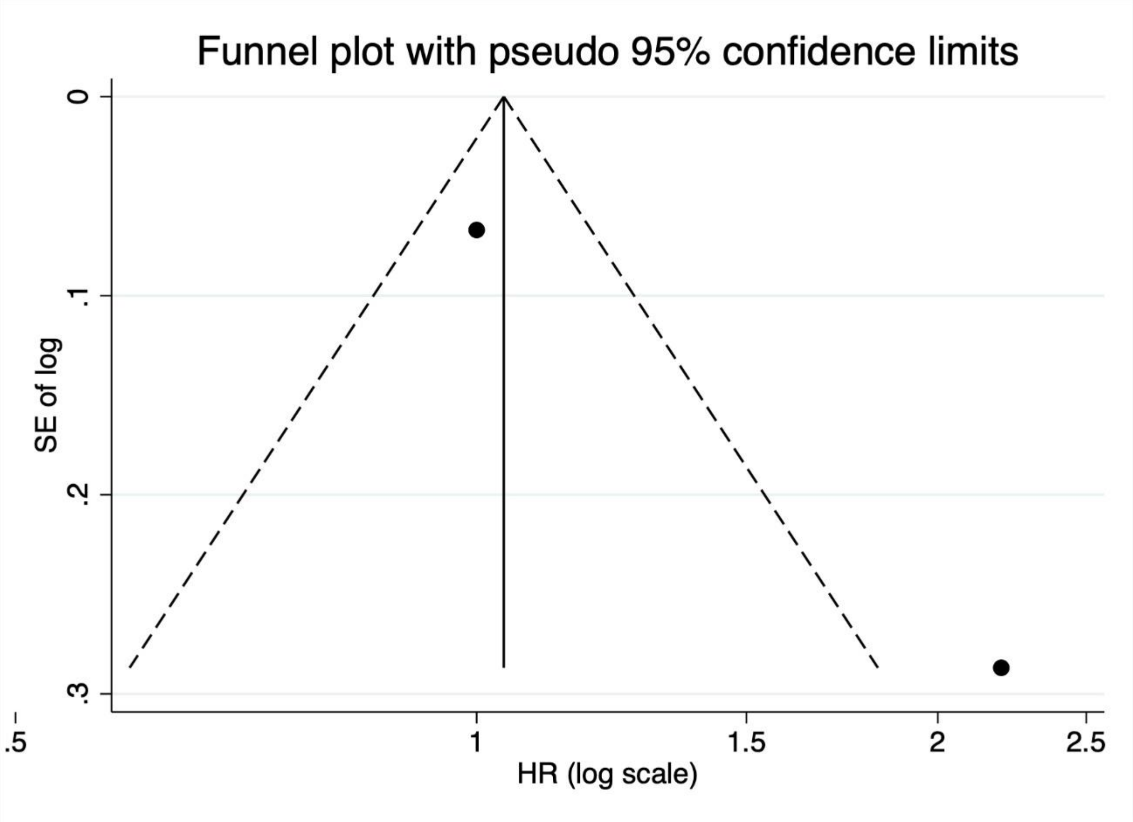

Figure S1.13. Funnel plot-publication bias: multivariate analysis of treatment with rituximab and OS at 2-10 years.

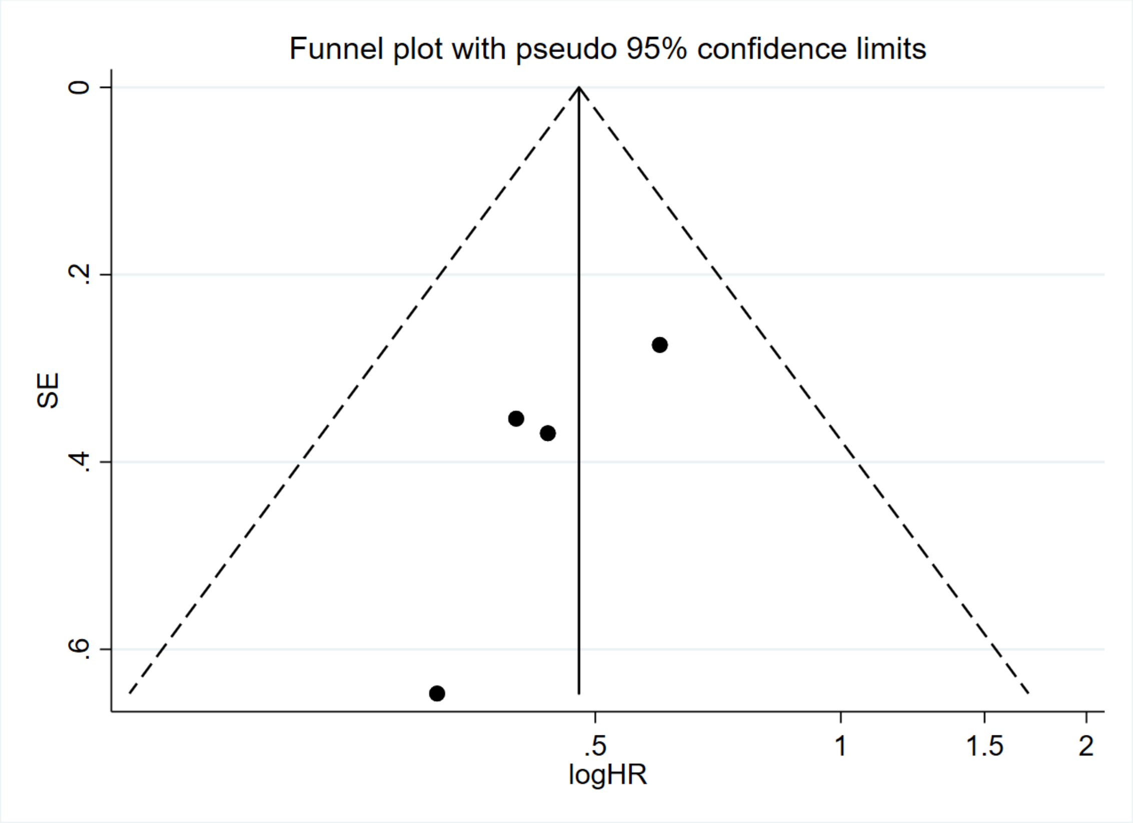

Figure S1.14. Funnel plot-publication bias: multivariate analysis of treatment with rituximab and PFS at 3-5 years.

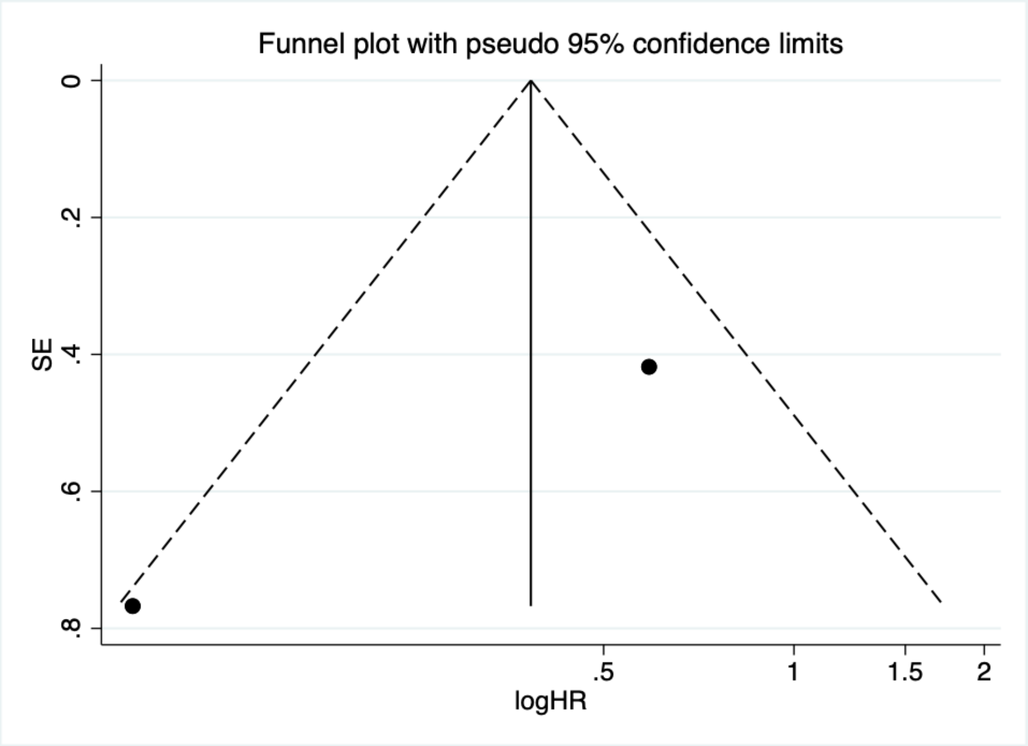

Supplement: Supplementary file 1 — Figure S1. [file CAM4-14-e70513-s003.pdf]
