# Supplementary material for: Prognostic Factors for Survival in Adults With Burkitt Lymphoma: A Systematic Review
Source: Cancer Med. 2025 Jan 29;14(3):e70513. doi: 10.1002/cam4.70513 (PMC11775923; doi:10.1002/cam4.70513)

Figure S2.1. Forest plot for the association between sex and overall survival at 5 years.

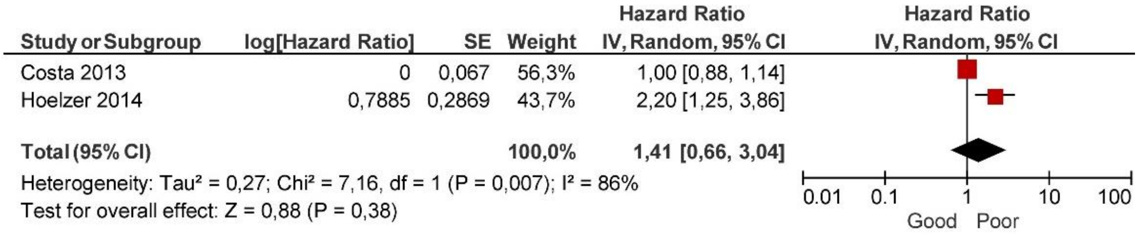

Figure S2.2. Forest plot for the association between bone marrow involvement and progression-free survival at 4-5 years.

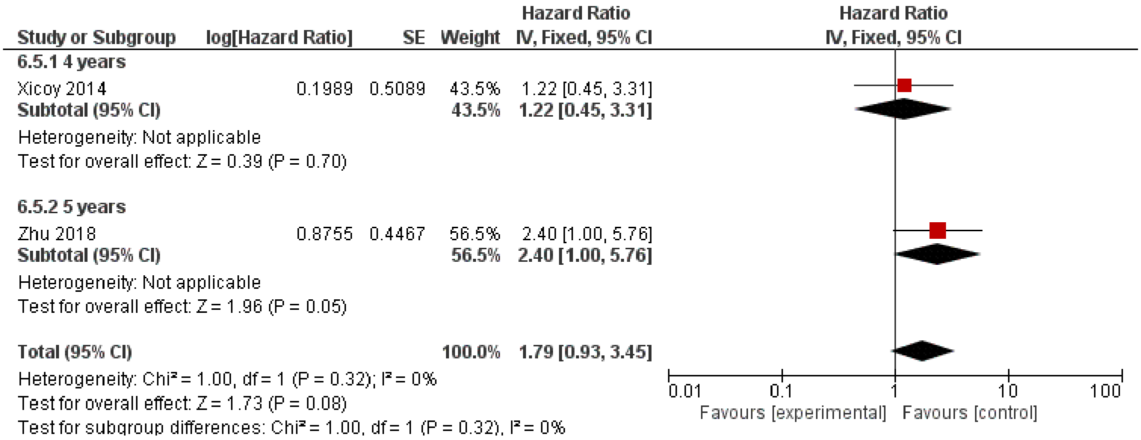

Supplement: Supplementary file 2 — Figure S2. [file CAM4-14-e70513-s007.pdf]
