# Supplementary material for: Prognostic Factors for Survival in Adults With Burkitt Lymphoma: A Systematic Review
Source: Cancer Med. 2025 Jan 29;14(3):e70513. doi: 10.1002/cam4.70513 (PMC11775923; doi:10.1002/cam4.70513)
Supplement: Supplementary file 3 — Table S1. [file CAM4-14-e70513-s004.docx]

| Supplementary Table S1. Search strategy |
| --- |
| Medline (Ovid platform) (February 22, 2022) |
| \| 1. Burkitt Lymphoma/ \| \| --- \| \| 1. burkitt.mp. \| \| 1. 1 or 2 \| \| 1. (child* or stepchild* or step-child* or kid or kids or girl or girls or boy or boys or teen* or youth* or youngster* or adolescent* or adolescence or preschool* or pre-school* or kindergarten* or school* or juvenile* or minors or p?ediatric* or PICU).ti,ab. or exp child/ \| \| 1. 3 not 4 \| \| 1. (immuno-histochemistry or immunohistochemistry).ti,ab. \| \| 1. flow cytometry.ti,ab. \| \| 1. conventional cytogenetic*.ti,ab. \| \| 1. molecular genetic*.ti,ab. \| \| 1. somatic mutation analysis.ti,ab. \| \| 1. Flow Cytometry/ \| \| 1. Biomarkers/ \| \| 1. Immunohistochemistry/ \| \| 1. Cytogenetics/ \| \| 1. Pathology/ \| \| 1. Molecular Biology/ \| \| 1. DNA Mutational Analysis/ \| \| 1. Risk Factors/ \| \| 1. ((kidney or liver or heart or lung or pancreas) and function).ti,ab. \| \| 1. Comorbidity/ \| \| 1. Bone Marrow Cells/ \| \| 1. bone marrow.ti,ab. \| \| 1. Central Nervous System/ \| \| 1. (central nervous system or CNS).ti,ab. \| \| 1. 6 or 7 or 8 or 9 or 10 or 11 or 12 or 13 or 14 or 15 or 16 or 17 or 18 or 19 or 20 or 21 or 22 or 23 or 24 \| \| 1. Cohort Studies/ \| \| 1. Incidence/ \| \| 1. Mortality/ \| \| 1. Follow-Up Studies/ \| \| 1. prognos*.tw. \| \| 1. predict*.tw. \| \| 1. course.tw. \| \| 1. Survival Analysis/ \| \| 1. 26 or 27 or 28 or 29 or 30 or 31 or 32 or 33 \| \| 1. 5 and 25 and 34 \| \| 1. limit 35 to (english language and humans) \| |
| EMBASE (Elsevier interface) (February 22, 2022) |
| 1. 'burkitt lymphoma'/exp 2. burkitt:ti,ab 3. #1 OR #2 4. OR p?ediatric*:ti,ab OR picu:ti,ab OR 'child'/exp OR kindergarten*:ti,ab OR school*:ti,ab OR juvenile*:ti,ab OR minors:ti,ab child*:ti,ab OR stepchild*:ti,ab OR 'step-child*':ti,ab OR kid:ti,ab OR kids:ti,ab OR girl:ti,ab OR girls:ti,ab OR boy:ti,ab OR boys:ti,ab OR teen*:ti,ab OR youth*:ti,ab OR youngster*:ti,ab OR adolescent*:ti,ab OR adolescence:ti,ab OR preschool*:ti,ab OR 'pre-school*':ti,ab 5. #3 NOT #4 6. 'immuno-histochemistry':ti,ab OR immunohistochemistry:ti,ab 7. 'flow cytometry':ti,ab 8. 'conventional cytogenetic*':ti,ab 9. 'molecular genetic*':ti,ab 10. 'somatic mutation analysis':ti,ab 11. 'flow cytometry'/mj 12. 'biological marker'/mj 13. 'immunohistochemistry'/mj 14. 'cytogenetics'/mj 15. 'pathology'/mj 16. 'molecular biology'/mj 17. 'dna mutational analysis'/exp 18. 'risk factor'/mj 19. (kidney:ti,ab OR liver:ti,ab OR heart:ti,ab OR lung:ti,ab OR pancreas:ti,ab) AND function:ti,ab 20. 'comorbidity'/mj 21. 'bone marrow cell'/exp 22. 'bone marrow':ti,ab 23. 'central nervous system'/mj 24. 'central nervous system':ti,ab OR cns:ti,ab 25. #6 OR #7 OR #8 OR #9 OR #10 OR #11 OR #12 OR #13 OR #14 OR #15 OR #16 OR #17 OR #18 OR #19 OR #20 OR #21 OR #22 OR #23 OR #24 26. 'cohort analysis'/mj 27. 'incidence'/mj 28. 'mortality'/mj 29. 'follow up'/mj 30. prognos*:ti,ab 31. predict*:ti,ab 32. course:ti,ab 33. 'survival analysis'/mj 34. #26 OR #27 OR #28 OR #29 OR #30 OR #31 OR #32 OR #33 35. #5 AND #25 AND #34 36. #5 AND #25 AND #34 AND [english]/lim AND [humans]/lim |
| Cochrane (CENTRAL) (February 22, 2022) |
| 1. MeSH descriptor: [Burkitt Lymphoma] explode all trees 2. burkitt 3. #1 or #2 4. (child* OR stepchild* OR step-child* OR kid OR kids OR girl OR girls OR boy OR boys OR teen* OR youth* OR youngster* OR adolescent* OR adolescence OR preschool* OR pre-school* OR kindergarten* OR school* OR juvenile* OR minors OR p?ediatric* OR PICU):ti,ab 5. MeSH descriptor: [Child] explode all trees 6. #4 or #5 7. #3 not #6 8. (immuno-histochemistry or immunohistochemistry):ti,ab 9. flow cytometry:ti,ab 10. conventional cytogenetic*:ti,ab 11. molecular genetic*:ti,ab 12. somatic mutation analysis:ti,ab 13. MeSH descriptor: [Flow Cytometry] explode all trees 14. MeSH descriptor: [Biomarkers] explode all trees 15. MeSH descriptor: [Immunohistochemistry] explode all trees 16. MeSH descriptor: [Cytogenetics] explode all trees 17. MeSH descriptor: [Pathology] explode all trees 18. MeSH descriptor: [Molecular Biology] explode all trees 19. MeSH descriptor: [DNA Mutational Analysis] explode all trees 20. MeSH descriptor: [Risk Factors] explode all trees 21. ((kidney or liver or heart or lung or pancreas) and function):ti,ab 22. MeSH descriptor: [Comorbidity] explode all trees 23. MeSH descriptor: [Bone Marrow Cells] explode all trees 24. bone marrow:ti,ab 25. MeSH descriptor: [Central Nervous System] explode all trees 26. (central nervous system or CNS):ti,ab 27. #8 or #9 or #10 or #11 or #12 or #13 or #14 or #15 or #16 or #17 or #18 or #19 or #20 or #21 or #22 or #23 or #24 or #25 or #26 28. #7 and #27 |
