# Supplementary material for: Prognostic Factors for Survival in Adults With Burkitt Lymphoma: A Systematic Review
Source: Cancer Med. 2025 Jan 29;14(3):e70513. doi: 10.1002/cam4.70513 (PMC11775923; doi:10.1002/cam4.70513)
Supplement: Supplementary file 4 — Table S2. [file CAM4-14-e70513-s005.docx]

| Supplementary Table S2. Excluded studies |
| --- |
| Abstract |
| 1. Alderuccio JP, Olszewski AJ, Evens AM, Collins GP, Danilov A, Bower M, et al. Prognostication, Survival and Treatment-Related Outcomes in HIV-Associated Burkitt Lymphoma (HIV-BL): A US and UK Collaborative Analysis. Blood. 2020;136((Alderuccio J.P.; Lossos I.S.) Sylvester Comprehensive Cancer Center, University of Miami School of Medicine, Miami, FL, United States):49–50. |
| 1. Allen A, Gill K, Hoehn D, Sulis M, Bhagat G, Alobeid B. C-myc protein expression in B-cell acute lymphoblastic leukemia, prognostic significance? Leuk Res. 2014 Sep;38(9):1061–6. |
| 1. Aukema SM, Theil L, Rohde M, Bauer B, Bradtke J, Burkhardt B, et al. Sequential karyotyping in Burkitt lymphoma reveals a linear clonal evolution with increase in karyotype complexity and a high frequency of recurrent secondary aberrations. Br J Haematol. 2015 Sep;170(6):814–25. |
| 1. Benevolo G, Pioltelli P, Spina M, Botto B, Stacchini A, Evangelista A, et al. In aggressive NHL patients the cases resulted as positive at cerebrospinal fluid flow cytometry analysis seems to have an higher risk of SNC relapse in comparison to patients FCM negative. Haematologica. 2010;95((Benevolo G.; Botto B.; Vitolo U.) Haematology 2 ASO San Giovanni Battista, Turin, Italy):S48. |
| 1. 1. Bobillo S, Abrisqueta P, Sánchez-González B, Giné E, Romero S, Alcoceba M, et al. Post-transplant monomorphic Burkitt’s lymphoma: Clinical characteristics and outcome of a multicenter series. Hematol Oncol. 2017;35((Bobillo S.; Abrisqueta P.; López A.; Bosch F.) Department of Hematology, Vall d’Hebron University Hospital, Barcelona, Spain):349–50. |
| 1. Boltezar L, Rozman S, Novakovic BJ. Survival of adult Burkitt lymphoma patients treated with the NHL-BFM 90 protocol in a 10-year period in Slovenia. Annals of Oncology. 2020 Sep 1;31:S653. |
| 1. Chihara D, Fowler NH, Oki Y, Fanale MA, Nastoupil L, Westin JR, et al. Difference in Survival Outcome of Primary Central Nervous System Lymphoma By Histologic Types. Blood. 2017 Dec 7;130(Supplement 1):4137. |
| 1. Clara J, Dalia S, Shah B, Bello C, Chervenick P, Salcedo LMJ, et al. Survival Impact of Serum Albumin (SA) in Patients with Acquired Immunodeficiency Syndrome (AIDS) Related Non-Hodgkin’s Lymphomas. Clinical Lymphoma, Myeloma and Leukemia. 2016 Sep 1;16:S106–7. |
| 1. Decker D, Egan PC, Treaba DO, Olszewski AJ. Survival and Risk of Central Nervous System Recurrence in Burkitt or High-Grade B-Cell Lymphoma in the DA-EPOCH-R Era. Blood. 2018 Nov 29;132(Supplement 1):2983. |
| 1. Del Galy AS, Kouatchet A, Mercier M, Hamel JF, Raveau T, Moles MP, et al. Neurologic and hepatic failures at ICU admission are associated with worst prognosis for patients with lymphomas. Ann Intensive Care [Internet]. 2016;6((Del Galy A.S., [aurelien.sutradelgaly@gmail](mailto:aurelien.sutradelgaly@gmail).com; Mercier M.; Moles M.-P.; Clavert A.; Hunault-Berger M.; Ifrah N.; Schmidt-Tanguy A.) Maladies Du Sang, Centre Hospitalier Universitaire d’Angers, Angers, France). Available from: <https://www>.embase.com/search/results?subaction=viewrecord&id=L72342924&from=export |
| 1. Evens AM, Danilov A, Jagadeesh D, Sperling A, Kim SH, Vaca R, et al. The Evaluation and Treatment (Tx) of Burkitt Lymphoma (BL) in the Modern Era: Real World (RW) Outcomes and Prognostication across 26 US Cancer Centers (CC). Blood. 2019 Nov 13;134(Supplement_1):397. |
| 1. Fujiwara S ichiro, Tatara R, Okazuka K, Oh I, Ohmine K, Suzuki T, et al. Profiles Of De Novo CD25-Positive Mature B-Cell Lymphomas. Blood. 2013 Nov 15;122(21):4308. |
| 1. Garrido T, Melo T, Andrade J, Guimarães J. Aids-related lymphomas (ARL), outcome and prognostic factors. Haematologica. 2013;98((Garrido T.; Melo T.; Andrade J.; Guimarães J.) Centro Hospitalar São João, Porto, Portugal):140. |
| 1. Gordon MJ, Danilov AV. Prognostic Markers in Cardiac Non-Hodgkin Lymphoma: A Retrospective Review. Blood. 2015 Dec 3;126(23):5007. |
| 1. Gupta S, Jain S, Sandhu S, Sreenivasappa S, Pattali S, Braik T, et al. A Retrospective Analysis of All Hematological Malignancies in Patients Infected with HIV, a Subset Analysis of the CHAMP Study (Cook County Hospital (CCH) AIDS Malignancy Project),. Blood. 2011 Nov 18;118(21):3693. |
| 1. Intermesoli T, Rossi G, Delaini F, Romani C, Pogliani E, Pagani C, et al. Cure rates and toxicity vary according to age < vs. > 55 years in B-ALL and burkitt lymphoma treated with the German chemotherapy plus rituximab protocol: Italian study on over 100 patients. Haematologica. 2011;96((Intermesoli T.) Ospedali Riuniti, Bergamo, Italy):239. |
| 1. Intermesoli T, Rambaldi A, Rossi G, Delaini F, Romani C, Pogliani EM, et al. High cure rates in Burkitt lymphoma and leukemia: a Northern Italy Leukemia Group study of the German short intensive rituximab-chemotherapy program. Haematologica. 2013 Nov;98(11):1718–25. |
| 1. Jiang M, Niu T. Clinical Research on Burkitt Lymphoma—a Single-Center Report in China. Blood. 2014 Dec 6;124(21):5461. |
| 1. Kojima Y, Hagiwara S, Yamamoto H, Uehira T, Ajisawa A, Kitanaka A, et al. Clinical Analysis and Treatment Outcome of Aids-Related Burkitt Lymphoma in Japan. Annals of Oncology. 2012 Oct 1;23:xi108. |
| 1. Latos-Grażyńska E, Kazanowska B, Wróbel G, Kulej D, Chybicka A. Analysis of treatment failure in patients treated for NHL-B /B-ALL im Polish Pediatric Leukemia/Lymphoma Study Group. Annals of Oncology [Internet]. 2011 [cited 2023 Apr 20];22(suppl.4). Available from: <https://ppm>.edu.pl/info/article/UMW67632ae904514ffca38f291787d6b99a/ |
| 1. Loghavi S, Xu-Monette ZY, Fayad L, Kwak LW, Dabaja BS, Medeiros LJ, et al. Clinical and Biological Features of Primary Testicular B-Cell Lymphoma – a Single-Institution Study of 89 Cases. Blood. 2014 Dec 6;124(21):3036. |
| 1. Lukina A, Baryakh E, Kravchenko S, Birukova L, Gemdjian E, Magomedova A, et al. Acute renal failure in burkitt’s lymphoma patients undergoing BL-M-04 treatment protocol. Haematologica. 2012;97((Lukina A.; Baryakh E.; Kravchenko S.; Birukova L.; Gemdjian E.; Magomedova A.; Kremenetskaya A.; Vorobiev A.) National Research Center For Hematology, Moscow, Russian Federation):664. |
| 1. Manji F, Chow E, Gerrie A, Chua N, Puckrin R, Stewart D, et al. Outcomes in Relapsed/Refractory Burkitt Lymphoma: A Multi-Centre Canadian Experience. Blood. 2021 Nov 5;138:2525–2525. |
| 1. Mathew BM, Dalia S, Hall J, Kuykendall A, Shah BD, Bello CM, et al. Analysis of prognostic factors in patients with HIV-associated aggressive B-cell non-Hodgkin lymphomas. JCO. 2014 May 20;32(15_suppl):e19512–e19512. |
| 1. Metke F, Jehn CF, Salwender H, Dahmash F, Lange-Husken C, Glas B, et al. Only advanced disease stage and extranodal involvement are risk factors for lymphomatous meningitis of Burkitt Lymphoma at time-point of diagnosis. Oncol Res Treat. 2018;41((Metke F.; Jehn C.-F.; Dahmash F.; Lange-Husken C.; Elmaagacli A.H.) AK St. Georg, Hamatologie, Hamburg, Germany):48. |
| 1. Nam A, Chadburn A. Expression of MYC and BCL2 in aids-related high grade B cell non-hodgkin lymphomas. Lab Invest. 2016;96((Nam A.; Chadburn A.) Weill Cornell Medical College-New York Presbyterian Hospital, New York, NY):364A. |
| 1. Niitsu N, Takahashi N, Kohri M, Asou N, Tamaru J ichi, Sakai J, et al. Clinicopathological Features and Prognosis of B-Cell Lymphoma, Unclassifiable, with Features Intermediate Between Diffuse Large B-Cell Lymphoma and Burkitt Lymphoma with t(14;18) and 8q24 Translocation in the Rituximab Era. Blood. 2014 Dec 6;124(21):1633. |
| 1. Nooruddin Z, Pan Z, Gross L, Weitzenkamp D, Pollyea D, Gutman J, et al. Final results of Ihc analysis and prognostic factors in post transplant lymphoproliferative disorders after solid organ transplantation. Haematologica. 2016;101((Nooruddin Z.; Pan Z.; Pollyea D.; Gutman J.; Robinson W.; Smith C.; Schowinsky J.; Haverkos B.; Purev E.; Sherbenou D.; Rabinovitch R.; Ney D.; Jordan C.T.; Kamdar M.) Blood Cancer and BMT, Denver, United States):276–7. |
| 1. Olszewski AJ, Chorzalska AD, Petersen M, Ollila TA, Zayac A, Kurt H, et al. Cerebrospinal Fluid (CSF) Analysis of Tumor-Specific Cell-Free DNA (cfDNA) As a Diagnostic and Prognostic Tool for Central Nervous System (CNS) Invasion in Lymphoma. Blood. 2020 Nov 5;136:21–2. |
| 1. Ribera JM, García O, Buendía-Ureña B, Terol MJ, Vicent A, Vall-Llovera F, et al. Validation of the Burkitt Lymphoma International Prognostic Index in patients treated with two prospective chemoimmunotherapy trials in Spain. Leuk Lymphoma. 2022 Aug;63(8):1993–6. |
| 1. Ribrag V, Koscielny S, Bouabdallah K, Salles G, Casasnovas O, Recher C, et al. Addition of Rituximab Improves Outcome of HIV Negative Patients with Burkitt Lymphoma Treated with the Lmba Protocol: Results of the Randomized Intergroup (GRAALL-Lysa) LMBA02 Protocol. (IGR sponsored LMBA02, NCT00180882). Blood. 2012 Nov 16;120(21):685. |
| 1. Rizzieri DA, Johnson JL, Byrd JC, Lozanski G, Powell BL, Shea TC, et al. Efficacy and Toxicity of Rituximab and Brief Duration, High Intensity Chemotherapy with Filgrastim Support for Burkitt or Burkitt – Like Leukemia/Lymphoma: Cancer and Leukemia Group B (Calgb) Study 10002. Blood. 2010 Nov 19;116(21):858. |
| 1. Saillard C, Etienne A, Charbonnier A, D’Incan E, Rey J, Chicouene A, et al. Acute lymphoblastic leukemia in the elderly: Prognostic factors and comorbidities impact. Haematologica. 2013;98((Saillard C.; Etienne A.; Charbonnier A.; D’Incan E.; Rey J.; Chicouene A.; Blaise D.; Vey N.; Prebet T.) Hematology, Marseille, France):262. |
| 1. Samra B, Richard-Carpentier G, Wierda W, Khoury J, Hussein SE, Ferrajoli A, et al. ALL-326: Low Incidence of Central Nervous System (CNS) Relapse with Hyper-CVAD-R Regimen in Adults with Burkitt Lymphoma/Leukemia (BL) and High-Grade B-Cell Lymphoma (HGBCL). Clinical Lymphoma, Myeloma and Leukemia. 2020 Sep 1;20:S170–1. |
| 1. Satou A, Asano N, Kato S, Elsayed AA, Nakamura N, Miyoshi H, et al. Prognostic Impact of MUM1/IRF4 Expression in Burkitt Lymphoma (BL): A Reappraisal of 88 BL Patients in Japan. Am J Surg Pathol. 2017 Mar;41(3):389–95. |
| 1. Short NJ, Kantarjian HM, Jabbour E, O’Brien S, Faderl S, Burger JA, et al. Persistence of Cytogenetic Abnormalities at Complete Remission Is Not Prognostic for Relapse-Free or Overall Survival in Adult Patients with Acute Lymphoblastic Leukemia. Blood. 2015 Dec 3;126(23):1416. |
| 1. Song JY, Venkataraman G, Fedoriw Y, Herrera AF, Siddiqi T, Alikhan MB, et al. Burkitt leukemia limited to the bone marrow has a better prognosis than Burkitt lymphoma with bone marrow involvement in adults. Leuk Lymphoma. 2016;57(4):866–71. |
| 1. Sýkorová A, Pytlík R, Móciková H, Janíková A, Procházka V, Belada D, et al. Burkitt lymphoma–ulticentre retrospective data analysis from the Czech Lymphoma Study Group–NiHiL project. Hematological Oncology. 2017;35(S2):342–3. |
| 1. Yamamoto H, Hagiwara S, Kojima Y, Uehira A, Ajisawa A, Kitanaka A, et al. Rituxiamb Did Not Improve Clinical Outcomes In AIDS-Related Burkitt Lymphoma. Blood. 2011 Nov 18;118(21):1629. |
| 1. Zayac A, Evens AM, Stadnik A, Smith SD, Jagadeesh D, Leslie LA, et al. Outcomes of Patients with Newly-Diagnosed Burkitt Lymphoma (BL) and Central Nervous System (CNS) Involvement Treated in the Modern Era: A Multi-Institutional Real-World Analysis. Blood. 2019 Nov 13;134(Supplement_1):402. |
| Outcome |
| 1. Albano D, Bosio G, Re A, Pagani C, Giubbini R, Bertagna F. Metabolic behavior and prognostic value of early and end of treatment 18F-FDG PET/CT in adult Burkitt’s lymphoma: the role of Deauville and IHP criteria. Leukemia & Lymphoma. 2019 Jan 28;60(2):326–33. 2. Galicier L, Fieschi C, Borie R, Meignin V, Daniel MT, Gérard L, et al. Intensive chemotherapy regimen (LMB86) for St Jude stage IV AIDS-related Burkitt lymphoma/leukemia: a prospective study. Blood. 2007 Oct 15;110(8):2846–54. |
| 1. Smyth L, Browne PV, Conneally E, Flynn C, Hayden P, Jeffers M, et al. Burkitt leukaemia/lymphoma: R-CODOX-M/R-IVAC remains gold standard treatment in BL. Ir J Med Sci. 2016 Nov;185(4):773–7. |
| Population |
| 1. Algrin C, Faguer S, Lemiale V, Lengliné E, Boutboul D, Amorim S, et al. Outcomes after intensive care unit admission of patients with newly diagnosed lymphoma. Leuk Lymphoma. 2015 May;56(5):1240–5. 2. Barta SK, Samuel MS, Xue X, Wang D, Lee JY, Mounier N, et al. Changes in the influence of lymphoma- and HIV-specific factors on outcomes in AIDS-related non-Hodgkin lymphoma. Ann Oncol. 2015 May;26(5):958–66. |
| 1. Bobillo S, Abrisqueta P, Sánchez-González B, Giné E, Romero S, Alcoceba M, et al. Posttransplant monomorphic Burkitt’s lymphoma: clinical characteristics and outcome of a ulticentre series. Ann Hematol. 2018 Dec;97(12):2417–24. |
| 1. Bürgesser MV, Gualco G, Diller A, Natkunam Y, Bacchi CE. Clinicopathological features of aggressive B-cell lymphomas including B-cell lymphoma, unclassifiable, with features intermediate between diffuse large B-cell and Burkitt lymphomas: a study of 44 patients from Argentina. Ann Diagn Pathol. 2013 Jun;17(3):250–5. |
| 1. Cardenas-Ortega A, Ramírez-Ibarguen AF, Rivera-Buendía F, Pérez-Jiménez C, Volkow-Fernández P, Martin-Onraet A. Clinical characteristics and outcomes of HIV positive patients with lymphoma in an oncological reference center in Mexico City. Medicine (Baltimore). 2020 Oct 2;99(40):e22408. |
| 1. Chang YY, Chang CH, Ku WW, Gau JP, Yu YB. Tumor lysis syndrome as a risk factor for very early mortality in HIV-associated non-Hodgkin’s lymphoma: A 10-year single-center experience. J Chin Med Assoc. 2020 Apr;83(4):371–6. |
| 1. Corazzelli G, Frigeri F, Russo F, Frairia C, Arcamone M, Esposito G, et al. RD-CODOX-M/IVAC with rituximab and intrathecal liposomal cytarabine in adult Burkitt lymphoma and ‘unclassifiable’ highly aggressive B-cell lymphoma. Br J Haematol. 2012 Jan;156(2):234–44. 2. Hong J, Kim SJ, Ahn JS, Song MK, Kim YR, Lee HS, et al. Treatment Outcomes of Rituximab Plus Hyper-CVAD in Korean Patients with Sporadic Burkitt or Burkitt-like Lymphoma: Results of a Multicenter Analysis. Cancer Res Treat. 2015 Apr;47(2):173–81. 3. Kasamon YL, Brodsky RA, Borowitz MJ, Ambinder RF, Crilley PA, Cho SY, et al. Brief intensive therapy for older adults with newly diagnosed Burkitt or atypical Burkitt lymphoma/leukemia. Leuk Lymphoma. 2013 Mar;54(3):483–90. |
| 1. Mead GM, Barrans SL, Qian W, Walewski J, Radford JA, Wolf M, et al. A prospective clinicopathologic study of dose-modified CODOX-M/IVAC in patients with sporadic Burkitt lymphoma defined using cytogenetic and immunophenotypic criteria (MRC/NCRI LY10 trial). Blood. 2008 Sep 15;112(6):2248–60. 2. Ribera JM, García O, Oriol A, Gil C, Montesinos P, Bernal T, et al. Feasibility and results of subtype-oriented protocols in older adults and fit elderly patients with acute lymphoblastic leukemia: Results of three prospective parallel trials from the PETHEMA group. Leuk Res. 2016 Feb;41:12–20. |
| 1. Samra B, Khoury JD, Morita K, Ravandi F, Richard-Carpentier G, Short NJ, et al. Long-term outcome of hyper-CVAD-R for Burkitt leukemia/lymphoma and high-grade B-cell lymphoma: focus on CNS relapse. Blood Adv. 2021 Oct 26;5(20):3913–8. |
| 1. Schommers P, Gillor D, Hentrich M, Wyen C, Wolf T, Oette M, et al. Incidence and risk factors for relapses in HIV-associated non-Hodgkin lymphoma as observed in the German HIV-related lymphoma cohort study. Haematologica. 2018 May;103(5):857–64. |
| 1. Schommers P, Hentrich M, Hoffmann C, Gillor D, Zoufaly A, Jensen B, et al. Survival of AIDS-related diffuse large B-cell lymphoma, Burkitt lymphoma, and plasmablastic lymphoma in the German HIV Lymphoma Cohort. British Journal of Haematology. 2015;168(6):806–10. |
| 1. Silva WF da, Garibaldi PMM, Rosa LI da, Bellesso M, Clé DV, Delamain MT, et al. Outcomes of HIV-associated Burkitt Lymphoma in Brazil: High treatment toxicity and refractoriness rates – A ulticentre cohort study. Leuk Res. 2020 Feb;89:106287. |
| 1. Song JY, Venkataraman G, Fedoriw Y, Herrera AF, Siddiqi T, Alikhan MB, et al. Burkitt leukemia limited to the bone marrow has a better prognosis than Burkitt lymphoma with bone marrow involvement in adults. Leuk Lymphoma. 2016;57(4):866–71. |
