# Supplementary material for: Prognostic Factors for Survival in Adults With Burkitt Lymphoma: A Systematic Review
Source: Cancer Med. 2025 Jan 29;14(3):e70513. doi: 10.1002/cam4.70513 (PMC11775923; doi:10.1002/cam4.70513)
Supplement: Supplementary file 5 — Table S3. [file CAM4-14-e70513-s002.docx]

**Supplementary Table S3: Risk of bias assessment for included studies (QUIPS tool)**

| **Study** | **Study Participation** | **Study Attrition** | **Prognostic Factor Measurement** | **Outcome Measurement** | **Study Confounding** | **Statistical Analysis and Reporting** | **Overall Risk of Bias** |
| --- | --- | --- | --- | --- | --- | --- | --- |
| Ahmed, 2021 [1] | Low | Low | Low | High | High | High | High |
| Albano, 2019 [2] | Low | Low | Low | High | High | High | High |
| Albano, 2019 [3] | Low | Low | Low | Low | High | High | High |
| Alderuccio, 2021^┼^ [4] | Low | Low | Low | Low | Low | Medium | Low |
| Barnes, 2011 [5] | Low | Low | Low | Low | Low | High | High |
| Castillo, 2013^£^ [6] | Medium | Low | Low | Low | High | Low | High |
| Chen, 2022 [7] | Low | Low | Low | Low | Medium | Medium | Medium |
| Chen, 2021 [8] | Medium | Low | Low | High | High | High | High |
| Choi, 2009 [9] | Low | Low | Low | Low | Medium | Medium | Medium |
| Costa, 2013^£^ [10] | High | Low | Low | Low | High | Medium | High |
| Evens, 2021^┼^ [11] | Low | Low | Low | Low | Low | Medium | Low |
| Forero-Castro, 2015 [12] | Low | Low | Low | High | High | Low | High |
| Ganesan, 2018 [13] | Low | Low | Low | Low | Low | Medium | Low |
| Hoelzer, 2014 [14] | Low | Low | Low | Low | Medium | Medium | Medium |
| Jakobsen, 2020 [15] | Low | Low | Low | Low | High | High | High |
| Jang, 2012 [16] | High | Medium | Medium | High | High | Low | High |
| Kim, 2021 [17] | Low | Low | Low | Low | High | Medium | High |
| Makoseh, 2021 [18] | High | Low | Low | Low | Low | Medium | High |
| Malkan, 2016 [19] | Low | Low | Low | Low | Low | High | High |
| Mukhtar, 2017^£^ [20] | Medium | Low | Low | Low | Low | High | High |
| Musekwa, 2020 [21] | Low | Low | Low | Low | High | High | High |
| Olszewski, 2021^┼^ [22] | Low | Low | Low | Low | High | Medium | High |
| Oriol, 2008 [23] | Low | Low | Low | Low | High | High | High |
| Phillips, 2020 [24] | Low | Low | Low | Low | High | High | High |
| Ribera, 2013 [25] | Low | Low | Low | Low | Low | Medium | Low |
| Ribrag, 2016 [26] | Low | Low | Low | Low | Low | Medium | Low |
| Rizzieri, 2014 [27] | Low | Low | Low | Low | High | High | High |
| Roschewski, 2020 [28] | Low | Low | Medium | Low | High | Low | High |
| Sakarou, 2019 [29] | Medium | Low | Low | Low | High | High | High |
| Tan, 2022 [30] | Low | Low | Low | Low | High | High | High |
| Wang, 2021 [31] | Low | Low | Low | Low | Low | Medium | Low |
| Wang, 2015 [32] | Low | Low | Low | Low | Low | Low | Low |
| Wästerlid, 2013 [33] | Low | Low | Low | Low | Medium | Low | Low |
| Wästerlid, 2011 [34] | Medium | Low | Low | Low | Medium | High | High |
| Wildes, 2014 [35] | Medium | Low | Medium | Low | Medium | Low | High |
| Xicoy, 2011 [36] | Low | Low | Low | Low | High | Low | High |
| Xicoy, 2014 [37] | Low | Low | Medium | Low | Medium | Low | Medium |
| Zayac, 2021^┼^ [38] | Low | Low | Low | Low | Low | High | High |
| Zhu, 2018 [39] | Low | Low | Low | Low | Low | Medium | Low |

Low: low risk of bias; Medium: Medium risk of bias; High: high risk of bias; ^┼^ Publications reporting analyses derived from the same sample; ^£^ Publications reporting analyses derived from the same database

**References**:

1. Ahmed A. The Outcomes of Sixty-Two Patients With Burkitt Lymphoma A Single-Center Study At King Abdulaziz Medical City Jeddah, Saudi Arabia. Int. J. Med. Sci. 2021 Mar 18;Volume 8.

2. Albano D, Bosio G, Pagani C, Re A, Tucci A, Giubbini R, et al. Prognostic role of baseline 18F-FDG PET/CT metabolic parameters in Burkitt lymphoma. Eur. J. Nucl. Med. Mol. Imaging. 2019 Jan 1;46(1):87–96.

3. Albano D, Re A, Tucci A, Giubbini R, Bertagna F. Prognostic role of ΔMTV and ΔTLG in Burkitt lymphoma. Ann. Nucl. Med. 2019 Apr;33(4):280–7.

4. Alderuccio JP, Olszewski AJ, Evens AM, Collins GP, Danilov AV, Bower M, et al. HIV-associated Burkitt lymphoma: outcomes from a US-UK collaborative analysis. Blood Adv. 2021 Jul 27;5(14):2852–62.

5. Barnes JA, Lacasce AS, Feng Y, Toomey CE, Neuberg D, Michaelson JS, et al. Evaluation of the addition of rituximab to CODOX-M/IVAC for Burkitt’s lymphoma: a retrospective analysis. Ann. Oncol. Off. J. Eur. Soc. Med. Oncol. 2011 Aug;22(8):1859–64.

6. Castillo JJ, Winer ES, Olszewski AJ. Population-based prognostic factors for survival in patients with Burkitt lymphoma: An analysis from the Surveillance, Epidemiology, and End Results database. Cancer. 2013;119(20):3672–9.

7. Chen M-T, Pan F, Chen Y-C, Zhang W, Lv H-J, Wang Z, et al. A novel prognostic index for sporadic Burkitt lymphoma in adult patients: a real-word multicenter study. BMC Cancer. 2022 Jan 7;22(1):45.

8. Chen M, Wang Z, Fang X, Yao Y, Ren Q, Chen Z, et al. Modified R-CODOX-M/IVAC chemotherapy regimens in Chinese patients with untreated sporadic Burkitt lymphoma. Cancer Biol. Med. 2021 Aug 15;18(3):833–40.

9. Choi MK, Jun HJ, Lee SY, Kim KH, Lim DH, Kim K, et al. Treatment outcome of adult patients with Burkitt lymphoma: results using the LMB protocol in Korea. Ann. Hematol. 2009 Nov;88(11):1099–106.

10. Costa LJ, Xavier AC, Wahlquist AE, Hill EG. Trends in survival of patients with Burkitt lymphoma/leukemia in the USA: an analysis of 3691 cases. Blood. 2013 Jun 13;121(24):4861–6.

11. Evens AM, Danilov A, Jagadeesh D, Sperling A, Kim S-H, Vaca R, et al. Burkitt lymphoma in the modern era: real-world outcomes and prognostication across 30 US cancer centers. Blood. 2021 Jan 21;137(3):374–86.

12. Forero-Castro M, Robledo C, Lumbreras E, Benito R, Hernández-Sánchez JM, Hernández-Sánchez M, et al. The presence of genomic imbalances is associated with poor outcome in patients with burkitt lymphoma treated with dose-intensive chemotherapy including rituximab. Br. J. Haematol. 2016 Feb;172(3):428–38.

13. Ganesan P, Ganesan TS, Atreya H, Kannan K, Radhakrishnan V, Dhanushkodi M, et al. DA-EPOCH-R in Aggressive CD 20 Positive B Cell Lymphomas: Real-World Experience. Indian J. Hematol. Blood Transfus. Off. J. Indian Soc. Hematol. Blood Transfus. 2018 Jul;34(3):454–9.

14. Hoelzer D, Walewski J, Döhner H, Viardot A, Hiddemann W, Spiekermann K, et al. Improved outcome of adult Burkitt lymphoma/leukemia with rituximab and chemotherapy: report of a large prospective multicenter trial. Blood. 2014 Dec 18;124(26):3870–9.

15. Jakobsen LH, Ellin F, Smeland KB, Wästerlid T, Christensen JH, Jørgensen JM, et al. Minimal relapse risk and early normalization of survival for patients with Burkitt lymphoma treated with intensive immunochemotherapy: an international study of 264 real‐world patients. Br. J. Haematol. 2020 May;189(4):661–71.

16. Jang S-J, Yoon DH, Kim S, Yoon S, Kim DY, Park C-S, et al. A unique pattern of extranodal involvement in Korean adults with sporadic Burkitt lymphoma: a single center experience. Ann. Hematol. 2012 Dec;91(12):1917–22.

17. Kim H-D, Cho H, Kim S, Lee K, Kang EH, Park JS, et al. Prognostic Stratification of Patients with Burkitt Lymphoma Using Serum β2-microglobulin Levels. Cancer Res. Treat. 2021 Jul;53(3):847–56.

18. Ma’koseh M, Amarin R, Tamimi F, Sharaf B, Abufara A, Shahin O, et al. Treatment of adult Burkitt lymphoma with the CALGB 1002 protocol: a single center experience in Jordan. Blood Res. 2021 Dec 31;56(4):279–84.

19. Malkan ÜY, Güneş G, Göker H, Haznedaroğlu İC, Acar K, Eliaçık E, et al. The Prognosis of Adult Burkitt’s Cell Leukemia in Real-Life Clinical Practice. Turk. J. Haematol. Off. J. Turk. Soc. Haematol. 2016 Dec 1;33(4):281–5.

20. Mukhtar F, Boffetta P, Risch HA, Park JY, Bubu OM, Womack L, et al. Survival predictors of Burkitt’s lymphoma in children, adults and elderly in the United States during 2000-2013. Int. J. Cancer. 2017 Apr 1;140(7):1494–502.

21. Musekwa E, Chapanduka ZC, Bassa F, Kruger M. An 8-year retrospective study of adult and paediatric Burkitt’s lymphoma at Tygerberg Hospital, South Africa. South Afr. J. Oncol. 2020 Apr 30;4(0):8.

22. Olszewski AJ, Jakobsen LH, Collins GP, Cwynarski K, Bachanova V, Blum KA, et al. Burkitt Lymphoma International Prognostic Index. J. Clin. Oncol. Off. J. Am. Soc. Clin. Oncol. 2021 Apr 1;39(10):1129–38.

23. Oriol A, Ribera J-M, Bergua J, Giménez Mesa E, Grande C, Esteve J, et al. High-dose chemotherapy and immunotherapy in adult Burkitt lymphoma: comparison of results in human immunodeficiency virus-infected and noninfected patients. Cancer. 2008 Jul 1;113(1):117–25.

24. Phillips EH, Burton C, Kirkwood AA, Barrans S, Lawrie A, Rule S, et al. Favourable outcomes for high‐risk Burkitt lymphoma patients (IPI 3‐5) treated with rituximab plus CODOX‐M/IVAC: Results of a phase 2 UK NCRI trial. EJHaem. 2020 Apr 29;1(1):133–41.

25. Ribera J-M, García O, Grande C, Esteve J, Oriol A, Bergua J, et al. Dose-intensive chemotherapy including rituximab in Burkitt’s leukemia or lymphoma regardless of human immunodeficiency virus infection status: final results of a phase 2 study (Burkimab). Cancer. 2013 May 1;119(9):1660–8.

26. Ribrag V, Koscielny S, Bosq J, Leguay T, Casasnovas O, Fornecker L-M, et al. Rituximab and dose-dense chemotherapy for adults with Burkitt’s lymphoma: a randomised, controlled, open-label, phase 3 trial. Lancet Lond. Engl. 2016 Jun 11;387(10036):2402–11.

27. Rizzieri DA, Johnson JL, Byrd JC, Lozanski G, Blum KA, Powell BL, et al. Improved efficacy using rituximab and brief duration, high intensity chemotherapy with filgrastim support for Burkitt or aggressive lymphomas: cancer and Leukemia Group B study 10 002. Br. J. Haematol. 2014 Apr;165(1):102–11.

28. Roschewski M, Dunleavy K, Abramson JS, Powell BL, Link BK, Patel P, et al. Multicenter Study of Risk-Adapted Therapy with Dose-Adjusted EPOCH-R in Adults with Untreated Burkitt Lymphoma. J. Clin. Oncol. 2020 May 26;38(22):2519–29.

29. Sakarou M, Eisele L, Dührsen U, Hüttmann A. Efficacy of the GMALL-B-ALL/NHL2002 protocol in Burkitt leukemia/lymphoma and aggressive non-Hodgkin-lymphomas with or without CNS involvement. Eur. J. Haematol. 2019 Mar;102(3):241–50.

30. Tan JY, Qiu TY, Chiang J, Tan YH, Yang VS, Chang EWY, et al. Burkitt lymphoma - no impact of HIV status on outcomes with rituximab-based chemoimmunotherapy. Leuk. Lymphoma. 2023 Mar;64(3):586–96.

31. Wang Z, Zhang R, Gong Z, Liu L, Shen Y, Chen J, et al. Real-world outcomes of AIDS-related Burkitt lymphoma: a retrospective study of 78 cases over a 10-year period. Int. J. Hematol. 2021 Jun;113(6):903–9.

32. Wang L, Wang H, Xia Z-J, Huang H-Q, Jiang W-Q, Lin T-Y, et al. Peripheral blood lymphocyte to monocyte ratio identifies high-risk adult patients with sporadic Burkitt lymphoma. Ann. Hematol. 2015 Oct;94(10):1645–54.

33. Wästerlid T, Brown PN, Hagberg O, Hagberg H, Pedersen LM, D’Amore F, et al. Impact of chemotherapy regimen and rituximab in adult Burkitt lymphoma: a retrospective population-based study from the Nordic Lymphoma Group. Ann. Oncol. Off. J. Eur. Soc. Med. Oncol. 2013 Jul;24(7):1879–86.

34. Wästerlid T, Jonsson B, Hagberg H, Jerkeman M. Population based study of prognostic factors and treatment in adult Burkitt lymphoma: a Swedish Lymphoma Registry study. Leuk. Lymphoma. 2011 Nov;52(11):2090–6.

35. Wildes TM, Farrington L, Yeung C, Harrington AM, Foyil KV, Liu J, et al. Rituximab is associated with improved survival in Burkitt lymphoma: a retrospective analysis from two US academic medical centers. Ther. Adv. Hematol. 2014 Feb;5(1):3–12.

36. Xicoy B, Ribera JM, Miralles P, La Cruz J, Oriol A, Valencia E, et al. Comparison of CHOP treatment with specific short-intensive chemotherapy in AIDS-related Burkitt’s lymphoma or leukemia. Med. Clin. (Barc.). 2011 Mar 26;136(8):323–8.

37. Xicoy B, Ribera J-M, Müller M, García O, Hoffmann C, Oriol A, et al. Dose-intensive chemotherapy including rituximab is highly effective but toxic in human immunodeficiency virus-infected patients with Burkitt lymphoma/leukemia: parallel study of 81 patients. Leuk. Lymphoma. 2014 Oct;55(10):2341–8.

38. Zayac AS, Evens AM, Danilov A, Smith SD, Jagadeesh D, Leslie LA, et al. Outcomes of Burkitt lymphoma with central nervous system involvement: evidence from a large multicenter cohort study. Haematologica. 2021 Jul 1;106(7):1932–42.

39. Zhu KY, Song KW, Connors JM, Leitch H, Barnett MJ, Ramadan K, et al. Excellent real-world outcomes of adults with Burkitt lymphoma treated with CODOX-M/IVAC plus or minus rituximab. Br. J. Haematol. 2018 Jun;181(6):782–90.
