# Supplementary material for: Prognostic Factors for Survival in Adults With Burkitt Lymphoma: A Systematic Review
Source: Cancer Med. 2025 Jan 29;14(3):e70513. doi: 10.1002/cam4.70513 (PMC11775923; doi:10.1002/cam4.70513)
Supplement: Supplementary file 6 — Table S4. [file CAM4-14-e70513-s006.docx]

Supplementary Table S4. Results of meta-analysis and subgroup analysis

| **PROGNOSTIC FACTOR /**  ***Variable*** | **Model** | **K** | **N** | **HR** | **95% CI** | **I^2^ (%)** | **Test for subgroup differences (%)**  **(P-value)** |
| --- | --- | --- | --- | --- | --- | --- | --- |
| **AGE** | | | | | | | |
| ***Overall survival (multivariate): 3-5 years*** | | | | | | | |
| *Subgroup analysis* | | | | | | | |
| >40 years | Fixed | 1 | 641 | 2.02 | 1.38, 2.96 | NA |  |
| >55 years | Fixed | 1 | 123 | 2.50 | 1.43, 4.39 | NA |  |
| >60 years | Fixed | 2 | 161 | 2.25 | 0.99, 5.07 | 0 |  |
| >65 years | Fixed | 1 | 81 | 4.03 | 1.36, 11.94 | NA |  |
| ***Progression-Free Survival (multivariate): 3-5 years*** | | | | | | | |
| *Subgroup analysis* | | | | | | | |
| >40 years | Fixed | 1 | 126 | 1.70 | 1.24, 2.33 | NA |  |
| >60 years | Fixed | 2 | 161 | 3.18 | 1.61, 6.29 | 0 |  |
| >65 years | Fixed | 1 | 81 | 2.43 | 0.98, 6.03 | NA |  |
| **SEX** | | | | | | | |
| ***Overall survival (multivariate): 5 years*** | | | | | | | |
| **Total** | Random | 2 | 2285 | 1.41 | 0.66, 3.04 | 86 | NA |
| ***Relative survival (multivariate): 5 years*** | | | | | | | |
| **Total** | Fixed | 1 | 2284 | 1.15 | 0.99, 1.34 | NA | NA |
| **RACE/ETHNICITY** | | | | | | | |
| ***Overall survival (multivariate): 5 years*** | | | | | | | |
| *Subgroup analysis* | | | | | | | |
| Black | Fixed | 1 | 1749 | 1.28 | 1.05, 1.56 | NA | NA |
| ***Relative survival (multivariate): 5 years*** | | | | | | | |
| **Total** | Random | 3 | 6086 | 1.10 | 0.84, 1.44 | 82 | 85.2 (0.001) |
| *Subgroup analysis* | | | | | | | |
| Black | Fixed | 1 | 1695 | 1.60 | 1.30, 1.97 | NA | NA |
| Hispanic | Fixed | 1 | 1887 | 1.08 | 0.90, 1.30 | NA | NA |
| Asian or  pacific  islander | Fixed | 1 | 2504 | 0.92 | 0.73, 1.16 | NA | NA |
| **HIV** | | | | | | | |
| ***Overall survival (multivariate)*** | | | | | | | |
| **Total** | Fixed | 2 | 191 | 1.53 | 0.55, 4.27 | 0 | 0 (0.90) |
| Subgroup analysis | | | | | | | |
| 3 years | Fixed | 1 | 80 | 1.58 | 0.50, 4.99 | NA |  |
| *5 years* | Fixed | 1 | 81 | 1.35 | 0.14, 13.02 | NA |  |
| ***Progression-free survival (multivariate)*** | | | | | | | |
| Subgroup analysis | | | | | | | |
| *5 years* | Fixed | 1 | 80 | 1.17 | (0.38, 3.60) | NA |  |
| **BONE MARROW INVOLVEMENT** | | | | | | | |
| ***Overall survival (multivariate)*** | | | | | | | |
| **Total** | Random | 5 | 847 | 1.69 | (1.07, 2.69) | 39 | 0 (0.55) |
| *Subgroup analysis* | | | | | | | |
| 2 years | Fixed | 1 | 64 | 1.96 | (0.72, 5.34) | NA |  |
| *4 years* | Fixed | 1 | 81 | 2.70 | (1.10, 6.64) | NA |  |
| *5 years* | Random | 3 | 702 | 1.46 | (0.76, 2.83) | 58 |  |
| ***Progression-free survival (multivariate):*** | | | | | | | |
| Total | Fixed | 2 | 162 | 1.79 | (0.93, 3.45) | 0 | 0 (0.32) |
| *Subgroup analysis* | | | | | | | |
| 4 years | Random | 1 | 81 | 1.22 | (0.45, 3.31) | NA |  |
| 5 years | Random | 1 | 81 | 2.40 | (1.00, 5.76) | NA |  |
| ***Relative survival (multivariate): 5 years*** | | | | | | | |
| **Total** | Random | 1 | 2751 | 1.25 | (0.99, 1.59) | NA |  |
| **CENTRAL NERVOUS INVOLVEMENT** | | | | | | | |
| ***Overall survival (multivariate):*** | | | | | | | |
| **Total** | Fixed | 3 | 979 | 1.71 | 1.25, 2.35 | 0 | 0 (0.55) |
| *Subgroup analysis* | | | | | | | |
| 2 years | Fixed | 1 | 258 | 1.40 | 0.60, 3.27 | NA |  |
| 3 years | Fixed | 2 | 721 | 1.77 | 1.26, 2.49 | 31 |  |
| ***Progression-free survival (multivariate): 3 years*** | | | | | | | |
| **Total** | Fixed | 2 | 721 | 1.61 | 1.15, 2.25 | 0 |  |
| **TREATMENT: RITUXIMAB** | | | | | | | |
| ***Overall survival (multivariate):*** | | | | | | | |
| **Total** | Fixed | 3 | 371 | 0.40 | 0.25, 0.64 | 0 | 0 (0.70) |
| *Subgroup analysis* | | | | | | | |
| 2 years | Fixed | 2 | 336 | 0.42 | 0.25, 0.69 | 0 |  |
| 10 years | Fixed | 1 | 35 | 0.32 | 0.19, 1.14 | NA |  |
| ***Progression-free survival (multivariate):*** | | | | | | | |
| **Total** | Random | 2 | 191 | 0.26 | 0.04, 1.60 | 78 | 78.4 (0.03) |
| *Subgroup analysis* | | | | | | | |
| 3 years | Random | 1 | 80 | 0.59 | 0.26, 1.34 | NA |  |
| 5 years | Random | 1 | 81 | 0.09 | 0.02, 0.41 | NA |  |
| **RISK STRATIFICATION** | | | | | | | |
| ***Overall survival (multivariate): 3 years*** | | | | | | | |
| **Total** | Fixed | 1 | 81 | 3.81 | (0.85, 17.08) | NA |  |
| **TREATMENT: METOTREXATE INCORPORATION** | | | | | | | |
| ***Overall survival (multivariate): 3 years*** | | | | | | | |
| **Total** | Fixed | 1 | 81 | 0.28 | 0.09, 0.87 | NA | NA |
| ***Progression-free survival (multivariate): 3 years*** | | | | | | | |
| **Total** | Fixed | 1 | 81 | 0.28 | 0.10, 0.78 | NA | NA |
| **PERFORMANCE STATUS (ECOG)** | | | | | | | |
| ***Overall survival (multivariate)*** | | | | | | | |
| **Total** | Random | 7 | 1417 | 2.79 | 1.84, 4.24 | 45 | 59.3 (0.04) |
| *Subgroup analysis* | | | | | | | |
| 2 years | Fixed | 2 | 415 | 2.54 | 1.61, 3.99 | 0 | NA |
| 3 years | Fixed | 1 | 641 | 1.80 | 1.24, 2.61 | NA | NA |
| 4 years | Fixed | 1 | 16 | 2.40 | 0.40, 14.40 | NA | NA |
| 5 years | Fixed | 2 | 310 | 3.92 | 1.93, 7.96 | NA | NA |
| 10 years | Fixed | 1 | 35 | 15.14 | 3.31, 69.24 | NA | NA |
| *Sensitivity analysis* | | | | | | | |
| *Removing* Wildes 2014 | Random | 6 | 1382 | 2.29 | 1.74. 3.01 | 3 | 25 (0.26) |
| ***Progression-free survival (multivariate):*** | | | | | | | |
| **Total** | Random | 3 | 738 | 2.38 | 1.17, 4.85 | 59 | 58.9 (0.09) |
| *Subgroup analysis* | | | | | | | |
| 3 years | Fixed | 1 | 641 | 1.60 | 1.13, 2.27 | NA | NA |
| 4 years | Fixed | 1 | 16 | 2.50 | 0.60, 10.42 | NA | NA |
| 5 years | Fixed | 1 | 81 | 4.35 | 1.89, 10.01 | NA | NA |
| **ALBUMIN** | | | | | | | |
| ***Overall survival (multivariate):*** | | | | | | | |
| **Total** | Fixed | 2 | 335 | 2.37 | 1.45, 3.87 | 0 | 0 (0.71) |
| *Subgroup analysis* | | | | | | | |
| 2 years | Fixed | 1 | 78 | 2.13 | 1.01, 4.48 | NA | NA |
| 3 years | Fixed | 1 | 257 | 2.56 | 1.33, 4.93 | NA | NA |

Note: HR: hazard ratio; NA: No apply; Random: Random effect
