# Supplementary material for: Prognostic Factors for Survival in Adults With Burkitt Lymphoma: A Systematic Review
Source: Cancer Med. 2025 Jan 29;14(3):e70513. doi: 10.1002/cam4.70513 (PMC11775923; doi:10.1002/cam4.70513)
Supplement: Supplementary file 7 — Table S5. [file CAM4-14-e70513-s001.docx]

Supplementary Table S5.1. Evidence profile for the prognostic effect of age in adults (≥18 years) with Burkitt´s lymphoma

| **No. of studies**  **(participants)** | **Study design** | **Certainty assessment domains** | | | | | | **Estimated effect size**  **HR (95% CI)** | **Certainty** |
| --- | --- | --- | --- | --- | --- | --- | --- | --- | --- |
|  |  | **Risk of bias** | **Inconsistency** | **Indirectness** | **Imprecision** | **Publication bias** | **Other considerations** |  |  |
| **Age >40 years overall survival** (multivariate): 3-5 years. | | | | | | | | | |
| 1 (408) [1] | Observational | Not serious | Not serious | Not serious^a^ | Not serious^b^ | Undetected^c,d^ | None | 2.02 (1.38, 2.96) | ⊕⊕⊕⊕  HIGH |
| **Age >55 years overall survival** (multivariate): 3-5 years. | | | | | | | | | |
| 1 (123) [2] | Observational | Not serious | Not serious | Not serious^a^ | Not serious^b^ | Undetected^c,d^ | None | 2.50 (1.43, 4.39) | ⊕⊕⊕⊕  HIGH |
| **Age >60 years overall survival** (multivariate): 3-5 years | | | | | | | | | |
| 2 (161) [3,4] | Observational | Serious^e^ | Not serious | Not serious^a^ | Not serious^b^ | Undetected^c,d^ | None | 3.92 (1.91, 8.05) | ⊕⊕⊕Ο  MODERATE |
| **Age >65 years overall survival** (multivariate): 3-5 years | | | | | | | | | |
| 1 (81) [5] | Observational | Serious^e^ | Not serious | Not serious^a^ | Not serious^b^ | Undetected^c,d^ | None | 3.02 (1.17, 7.80) | ⊕⊕⊕Ο  MODERATE |
| **Age >40 years progression-free survival** (multivariate): 3-5 years | | | | | | | | | |
| 1 (408) [1] | Observational | Not serious | Not serious | Not serious^a^ | Not serious^b^ | Undetected^c,d^ | None | 1.70 (1.24, 2.33) | ⊕⊕⊕⊕  HIGH |
| **Age >60 years progression-free survival** (multivariate): 3-5 years | | | | | | | | | |
| 2 (161) [3,4] | Observational | Serious^e^ | Not serious | Not serious^a^ | Not serious^b^ | Undetected^c,d^ | None | 3.18 (1.61, 6.29) | ⊕⊕⊕Ο  MODERATE |
| **Age >65 years progression-free survival** (multivariate): 3-5 years | | | | | | | | | |
| 1 (81) [5] | Observational | Serious^e^ | Not serious | Not serious^a^ | Serious^b^ | Undetected^c,d^ | None | 2.43 (0.98, 6.03) | ⊕⊕ΟΟ  LOW |

a Patients with Burkitt’s lymphoma, Burkitt's leukemia and Sporadic Burkitt’s lymphoma.
b Imprecision was defined by wide confidence intervals. It was rated down when the 95%CI included the null effect and appreciable risk and protective values.
c Publication bias was assessed by visual inspection of funnel plot asymmetry.
d Potential risk of publication bias was detected but no downgrading was performed because a higher number of publications is needed for a conclusive decision.
e The proportion of information from studies with a high risk of bias is sufficient to affect the interpretation of the results.

Table S5.2. Evidence profile for the prognostic effect of sex in adults (≥18 years) with Burkitt´s lymphoma

| **No. of studies**  **(participants)** | **Study design** | **Certainty assessment domains** | | | | | | **Estimated effect size**  **HR (95% CI)** | **Certainty** |
| --- | --- | --- | --- | --- | --- | --- | --- | --- | --- |
|  |  | **Risk of bias** | **Inconsistency** | **Indirectness** | **Imprecision** | **Publication bias** | **Other considerations** |  |  |
| **Overall survival** (multivariate): 5 years | | | | | | | | | |
| 2 (2285) [2,6] | Observational | Serious^a^ | Serious^b^ | Not serious^c^ | Serious^d^ | Undetected^e^ | None | 1.41 (0.66, 3.04) | ⊕◯◯◯  VERY LOW |
| **Relative survival** (multivariate): 5 years | | | | | | | | | |
| 1 (2284) [7] | Observational | Serious^f^ | Not serious | Not serious^c^ | Serious^d^ | Undetected^e^ | None | 1.15 (0.99, 1.34) | ⊕⊕◯◯  LOW |

a All the information came from studies with a medium or high risk of bias.
b High heterogeneity: I^2^=86% (p=0.007).
c Patients with Burkitt’s lymphoma.
d Imprecision was defined by wide confidence intervals. It was rated down when the 95%CI included the null effect and appreciable risk and protective values.
e Publication bias was assessed by visual inspection of funnel plot asymmetry.
f The only study included was rated as having an overall high risk of bias.

Table S5.3. Evidence profile for the prognostic effect of race/ethnicity in adults (≥18 years) with Burkitt´s lymphoma

| **No. of studies**  **(participants)** | **Study design** | **Certainty assessment domains** | | | | | | **Estimated effect size**  **HR (95% CI)** | **Certainty** |
| --- | --- | --- | --- | --- | --- | --- | --- | --- | --- |
|  |  | **Risk of bias** | **Inconsistency** | **Indirectness** | **Imprecision** | **Publication bias** | **Other considerations** |  |  |
| **Black race/ethnicity - Overall survival** (multivariate): 5 years | | | | | | | | | |
| 1 (1749) [6] | Observational | Serious^a^ | Not serious | Not serious^b^ | Not serious^c^ | Undetected^d^ | None | 1.28 (1.05, 1.56) | ⊕⊕⊕◯  MODERATE |
| **Black race/ethnicity - Relative survival** (multivariate): 5 years | | | | | | | | | |
| 1 (1695) [7] | Observational | Serious^a^ | Not serious | Not serious^b^ | Not serious^c^ | Undetected^d^ | None | 1.60 (1.30, 1.97) | ⊕⊕⊕◯  MODERATE |
| **Hispanic race/ethnicity - Relative survival** (multivariate): 5 years | | | | | | | | | |
| 1 (1887) [7] | Observational | Serious^a^ | Not serious | Not serious^b^ | Serious^c^ | Undetected^d^ | None | 1.08 (0.90, 1.30) | ⊕⊕◯◯  LOW |
| **Asian or pacific islander race/ethnicity - Relative survival** (multivariate): 5 years | | | | | | | | | |
| 1 (2504) [8] | Observational | Serious^a^ | Not serious | Not serious^b^ | Serious^c^ | Undetected^d^ | None | 0.92 (0.73, 1.16) | ⊕⊕◯◯  LOW |

a The only study included was rated as having an overall high risk of bias.
b Patients with Burkitt’s lymphoma.
c Imprecision was defined by wide confidence intervals. It was rated down when the 95%CI included the null effect and appreciable risk and protective values.
d Publication bias was assessed by visual inspection of funnel plot asymmetry.

Table S5.4. Evidence profile for the prognostic effect of HIV in adults (≥18 years) with Burkitt´s lymphoma

| **No. of studies**  **(participants)** | **Study design** | **Certainty assessment domains** | | | | | | **Estimated effect size**  **HR (95% CI)** | **Certainty** |
| --- | --- | --- | --- | --- | --- | --- | --- | --- | --- |
|  |  | **Risk of bias** | **Inconsistency** | **Indirectness** | **Imprecision** | **Publication bias** | **Other considerations** |  |  |
| **Overall survival** (multivariate): 3-5 years | | | | | | | | | |
| 2 (161) [3,4] | Observational | Serious^a^ | Not serious | Not serious^b^ | Serious^c^ | Undetected^d^ | None | 1.53 (0.55, 4.27) | ⊕⊕◯ ◯  LOW |
| **Progression-free survival** (multivariate): 3 years | | | | | | | | | |
| 1 (80) [3] | Observational | Serious^e^ | Not serious | Not serious^b^ | Serious^c^ | Undetected^d^ | None | 1.17 (0.38, 3.60) | ⊕⊕◯◯  LOW |

a The proportion of information from studies with a medium or high risk of bias is sufficient to affect the interpretation of the results.
b Patients with Burkitt’s lymphoma.
c Imprecision was defined by wide confidence intervals. It was rated down when the 95%CI included the null effect and appreciable risk and protective values.
d Publication bias was assessed by visual inspection of funnel plot asymmetry.
e The only study included was rated as having an overall high risk of bias.

Table S5.5. Evidence profile for the prognostic effect of performance status in adults (≥18 years) with Burkitt´s lymphoma

| **No. of studies**  **(participants)** | **Study design** | **Certainty assessment domains** | | | | | | **Estimated effect size**  **HR (95% CI)** | **Certainty** |
| --- | --- | --- | --- | --- | --- | --- | --- | --- | --- |
|  |  | **Risk of bias** | **Inconsistency** | **Indirectness** | **Imprecision** | **Publication bias** | **Other considerations** |  |  |
| **Overall survival** (multivariate): 2-10 years | | | | | | | | | |
| 7 (1417) [1,5,9–13] | Observational | Serious^a^ | Serious^b^ | Not serious^c^ | Not serious^d^ | Undetected^e,f^ | None | 2.79 (1.84, 4.24) | ⊕⊕◯◯  LOW |
| **Progression-free survival** (multivariate): 3-5 years | | | | | | | | | |
| 3 (738) [1,5,11] | Observational | Serious^a^ | Serious^g^ | Not seriou^c^ | Not serious^d^ | Undetected^e^ | None | 2.38 (1.17, 4.85) | ⊕⊕◯◯  LOW |

a The proportion of information from studies with a medium or high risk of bias is sufficient to affect the interpretation of the results.
b Moderate heterogeneity: I^2^ = 45% (p=0.09).
c Patients with Burkitt’s lymphoma (including Burkitt’s leukemia).
d Imprecision was defined by wide confidence intervals. It was rated down when the 95%CI included the null effect and appreciable risk and protective values.
e Publication bias was assessed by visual inspection of funnel plot asymmetry.
f Potential risk of publication bias was detected but no downgrading was performed because a higher number of publications is needed for a conclusive decision.
g Moderate heterogeneity: I^2^ = 59% (p=0.09).

Table S5.6. Evidence profile for the prognostic effect of risk stratification in adults (≥18 years) with Burkitt´s lymphoma

| **No. of studies**  **(participants)** | **Study design** | **Certainty assessment domains** | | | | | | **Estimated effect size**  **HR (95% CI)** | **Certainty** |
| --- | --- | --- | --- | --- | --- | --- | --- | --- | --- |
|  |  | **Risk of bias** | **Inconsistency** | **Indirectness** | **Imprecision** | **Publication bias** | **Other considerations** |  |  |
| **Overall survival** (multivariate): 3 years | | | | | | | | | |
| 1 (80) [3] | Observational | Serious^a^ | Not serious | Not serious^b^ | Serious^c^ | Undetected^d^ | None | 3.81 (0.85, 17.08) | ⊕⊕◯◯  LOW |

a The overall risk of bias was high.
b Patients with Burkitt’s lymphoma.
c Imprecision was defined by wide confidence intervals. It was rated down when the 95%CI included the null effect and appreciable risk and protective values.
d Publication bias was assessed by visual inspection of funnel plot asymmetry.

Table S5.7. Evidence profile for the prognostic effect of bone marrow involvement in adults (≥18 years) with Burkitt´s lymphoma

| **No. of studies**  **(participants)** | **Study design** | **Certainty assessment domains** | | | | | | **Estimated effect size**  **HR (95% CI)** | **Certainty** |
| --- | --- | --- | --- | --- | --- | --- | --- | --- | --- |
|  |  | **Risk of bias** | **Inconsistency** | **Indirectness** | **Imprecision** | **Publication bias** | **Other considerations** |  |  |
| **Overall survival** (multivariate): 2-5 years | | | | | | | | | |
| 5 (847) [2,4,10,14,15] | Observational | Serious^a^ | Not serious | Not serious^b^ | Not serious | Undetected^c^ | None | 1.69 (1.07, 2.69) | ⊕⊕⊕◯  MODERATE |
| **Progression-free survival** (multivariate): 4-5 years | | | | | | | | | |
| 2 (162) [4,15] | Observational | Serious^a^ | Not serious | Not serious^b^ | Serious^d^ | Undetected^c^ | None | 1.79 (0.93, 3.45) | ⊕⊕◯◯  LOW |
| **Relative survival** (multivariate): 5 years | | | | | | | | | |
| 1 (2751) [8] | Observational | Serious^a^ | Not serious | Not serious^b^ | Serious^d^ | Undetected^c^ | None | 1.25 (0.99, 1.59) | ⊕⊕◯◯  LOW |

a The proportion of information from studies with a medium or high risk of bias is sufficient to affect the interpretation of the results.
b Patients with Burkitt’s lymphoma, Burkitt's leukemia and sporadic Burkitt’s lymphoma.
c Publication bias was assessed by visual inspection of funnel plot asymmetry.
d Imprecision was defined by wide confidence intervals. It was rated down when the 95%CI included the null effect and appreciable risk and protective values.

Table S5.8. Evidence profile for the prognostic effect of central nervous system involvement in adults (≥18 years) with Burkitt´s lymphoma

| **No. of studies**  **(participants)** | **Study design** | **Certainty assessment domains** | | | | | | **Estimated effect size**  **HR (95% CI)** | **Certainty** |
| --- | --- | --- | --- | --- | --- | --- | --- | --- | --- |
|  |  | **Risk of bias** | **Inconsistency** | **Indirectness** | **Imprecision** | **Publication bias** | **Other considerations** |  |  |
| **Overall survival** (multivariate): 2-3 years | | | | | | | | | |
| 3 (979) [1,3,10] | Observational | Not serious^a^ | Not serious | Not serious^b^ | Not serious^c^ | Undetected^d^ | None | 1.71 (1.25, 2.35) | ⊕⊕⊕⊕  HIGH |
| **Progression-free survival** (multivariate): 3 years | | | | | | | | | |
| 2 (721) [1,3] | Observational | Not serious^a^ | Not serious | Not serious^e^ | Not serious^c^ | Undetected^d^ | None | 1.61 (1.15, 2.25) | ⊕⊕⊕⊕  HIGH |

a Most informations came from studies with a low risk of bias.
b Only patients Burkitt’s lymphoma (including Burkitt’s leukemia).
c Imprecision was defined by wide confidence intervals. It was rated down when the 95%CI included the null effect and appreciable risk and protective values.
d Publication bias was assessed by visual inspection of funnel plot asymmetry.
e Only patients with Burkitt’s lymphoma.

Table S5.9. Evidence profile for the prognostic effect of albumin in adults (≥18 years) with Burkitt´s lymphoma

| **No. of studies**  **(participants)** | **Study design** | **Certainty assessment domains** | | | | | | **Estimated effect size**  **HR (95% CI)** | **Certainty** |
| --- | --- | --- | --- | --- | --- | --- | --- | --- | --- |
|  |  | **Risk of bias** | **Inconsistency** | **Indirectness** | **Imprecision** | **Publication bias** | **Other considerations** |  |  |
| **Overall survival** (multivariate): 2-3 years | | | | | | | | | |
| 2 (335) [16,17] | RCT and observational | Not serious | Not serious | Not serious^a^ | Not serious^b^ | Undetected^c^ | None | 2.37 (1.45, 3.87) | ⊕⊕⊕⊕  HIGH |

a Patients with Burkitt’s lymphoma.
b Imprecision was defined by wide confidence intervals, was rated down when the 95%CI includes the null effect and appreciable risk and protective values.
c Publication bias was evaluated by visual assessment of funnel plot asymmetry.

Table S5.10. Evidence profile for the prognostic effect of treatment with rituximab in adults (≥18 years) with Burkitt´s lymphoma

| **No. of studies**  **(participants)** | **Study design** | **Certainty assessment domains** | | | | | | **Estimated effect size**  **HR (95% CI)** | **Certainty** |
| --- | --- | --- | --- | --- | --- | --- | --- | --- | --- |
|  |  | **Risk of bias** | **Inconsistency** | **Indirectness** | **Imprecision** | **Publication bias** | **Other considerations** |  |  |
| **Overall survival** (multivariate): 2-10 years | | | | | | | | | |
| 3 (371) [10,13,18] | Observational | Not serious^a^ | Not serious | Not serious^b^ | Not serious^c^ | Undetected^d^ | None | 0.40 (0.25, 0.64) | ⊕⊕⊕⊕  HIGH |
| **Progression-free survival** (multivariate): 3-5 years | | | | | | | | | |
| 2 (191) [3,4] | Observational | Serious^e^ | Serious^f^ | Not serious^b^ | Serious^c^ | Undetected^d^ | None | 0.26 (0.04, 1.60) | ⊕◯◯◯  VERY LOW |

a Most information came from studies with a low risk of bias.
b Patients with Burkitt’s lymphoma (including Burkitt’s leukemia).
c Imprecision was defined by wide confidence intervals. It was rated down when the 95%CI included the null effect and appreciable risk and protective values.
d Publication bias was assessed by visual inspection of funnel plot asymmetry.
e The proportion of information from studies with a medium or high risk of bias is sufficient to affect the interpretation of the results.

f High heterogeneity: I^2^ = 78% (p=0.03).

Table S5.11. Evidence profile for the prognostic effect of treatment with methotrexate in adults (≥18 years) with Burkitt´s lymphoma

| **No. of studies**  **(participants)** | **Study design** | **Certainty assessment domains** | | | | | | **Estimated effect size**  **HR (95% CI)** | **Certainty** |
| --- | --- | --- | --- | --- | --- | --- | --- | --- | --- |
|  |  | **Risk of bias** | **Inconsistency** | **Indirectness** | **Imprecision** | **Publication bias** | **Other considerations** |  |  |
| **Overall survival** (multivariate): 3 years | | | | | | | | | |
| 1 (81) [4] | Observational | Not serious | Not serious | Not serious^a^ | Not serious^b^ | Undetected^c^ | None | 0.28 (0.09, 0.87) | ⊕⊕⊕⊕  HIGH |
| **Progression-free survival** (multivariate): 3 years | | | | | | | | | |
| 1 (81) [4] | Observational | Not serious | Not serious | Not serious^b^ | Not serious^b^ | Undetected^c^ | None | 0.28 (0.10, 0.78) | ⊕⊕⊕⊕  HIGH |

a Patients with Burkitt’s lymphoma.
b Imprecision was defined by wide confidence intervals. It was rated down when the 95%CI included the null effect and appreciable risk and protective values.
c Publication bias was assessed by visual inspection of funnel plot asymmetry.

1. Evens AM, Danilov A, Jagadeesh D, Sperling A, Kim S-H, Vaca R, et al. Burkitt lymphoma in the modern era: real-world outcomes and prognostication across 30 US cancer centers. Blood. 2021 Jan 21;137(3):374–86.

2. Hoelzer D, Walewski J, Döhner H, Viardot A, Hiddemann W, Spiekermann K, et al. Improved outcome of adult Burkitt lymphoma/leukemia with rituximab and chemotherapy: report of a large prospective multicenter trial. Blood. 2014 Dec 18;124(26):3870–9.

3. Barnes JA, Lacasce AS, Feng Y, Toomey CE, Neuberg D, Michaelson JS, et al. Evaluation of the addition of rituximab to CODOX-M/IVAC for Burkitt’s lymphoma: a retrospective analysis. Ann Oncol. 2011 Aug;22(8):1859–64.

4. Zhu KY, Song KW, Connors JM, Leitch H, Barnett MJ, Ramadan K, et al. Excellent real-world outcomes of adults with Burkitt lymphoma treated with CODOX-M/IVAC plus or minus rituximab. Br J Haematol. 2018 Jun;181(6):782–90.

5. Kim H-D, Cho H, Kim S, Lee K, Kang EH, Park JS, et al. Prognostic Stratification of Patients with Burkitt Lymphoma Using Serum β2-microglobulin Levels. Cancer Res Treat. 2021 Jul;53(3):847–56.

6. Costa LJ, Xavier AC, Wahlquist AE, Hill EG. Trends in survival of patients with Burkitt lymphoma/leukemia in the USA: an analysis of 3691 cases. Blood. 2013 Jun 13;121(24):4861–6.

7. Castillo JJ, Winer ES, Olszewski AJ. Population-based prognostic factors for survival in patients with Burkitt lymphoma: An analysis from the Surveillance, Epidemiology, and End Results database. Cancer. 2013;119(20):3672–9.

8. Mukhtar F, Boffetta P, Risch HA, Park JY, Bubu OM, Womack L, et al. Survival predictors of Burkitt’s lymphoma in children, adults and elderly in the United States during 2000-2013. Int J Cancer. 2017 Apr 1;140(7):1494–502.

9. Wästerlid T, Jonsson B, Hagberg H, Jerkeman M. Population based study of prognostic factors and treatment in adult Burkitt lymphoma: a Swedish Lymphoma Registry study. Leuk Lymphoma. 2011 Nov;52(11):2090–6.

10. Wästerlid T, Brown PN, Hagberg O, Hagberg H, Pedersen LM, D’Amore F, et al. Impact of chemotherapy regimen and rituximab in adult Burkitt lymphoma: a retrospective population-based study from the Nordic Lymphoma Group. Ann Oncol. 2013 Jul;24(7):1879–86.

11. Forero-Castro M, Robledo C, Lumbreras E, Benito R, Hernández-Sánchez JM, Hernández-Sánchez M, et al. The presence of genomic imbalances is associated with poor outcome in patients with burkitt lymphoma treated with dose-intensive chemotherapy including rituximab. Br J Haematol. 2016 Feb;172(3):428–38.

12. Chen M-T, Pan F, Chen Y-C, Zhang W, Lv H-J, Wang Z, et al. A novel prognostic index for sporadic Burkitt lymphoma in adult patients: a real-word multicenter study. BMC Cancer. 2022 Jan 7;22(1):45.

13. Wildes TM, Farrington L, Yeung C, Harrington AM, Foyil KV, Liu J, et al. Rituximab is associated with improved survival in Burkitt lymphoma: a retrospective analysis from two US academic medical centers. Ther Adv Hematol. 2014 Feb;5(1):3–12.

14. Jang S-J, Yoon DH, Kim S, Yoon S, Kim DY, Park C-S, et al. A unique pattern of extranodal involvement in Korean adults with sporadic Burkitt lymphoma: a single center experience. Ann Hematol. 2012 Dec;91(12):1917–22.

15. Xicoy B, Ribera J-M, Müller M, García O, Hoffmann C, Oriol A, et al. Dose-intensive chemotherapy including rituximab is highly effective but toxic in human immunodeficiency virus-infected patients with Burkitt lymphoma/leukemia: parallel study of 81 patients. Leuk Lymphoma. 2014 Oct;55(10):2341–8.

16. Ribrag V, Koscielny S, Bosq J, Leguay T, Casasnovas O, Fornecker LM, et al. Rituximab and dose-dense chemotherapy for adults with Burkitt’s lymphoma: a randomised, controlled, open-label, phase 3 trial. The Lancet. 2016;387(10036).

17. Wang Z, Zhang R, Gong Z, Liu L, Shen Y, Chen J, et al. Real-world outcomes of AIDS-related Burkitt lymphoma: a retrospective study of 78 cases over a 10-year period. International Journal of Hematology. 2021;113(6).

18. Wang Z, Zhang R, Gong Z, Liu L, Shen Y, Chen J, et al. Real-world outcomes of AIDS-related Burkitt lymphoma: a retrospective study of 78 cases over a 10-year period. Int J Hematol. 2021 Jun;113(6):903–9.
